# Increased adiposity, inflammation, metabolic disruption and dyslipidemia in adult male offspring of DOSS treated C57BL/6 dams

**DOI:** 10.1038/s41598-018-38383-9

**Published:** 2019-02-06

**Authors:** Alexis M. Temkin, Robert R. Bowers, Candice Z. Ulmer, Kayla Penta, John A. Bowden, Jennifer Nyland, John E. Baatz, Demetri D. Spyropoulos

**Affiliations:** 1 Molecular and Cellular Biology and Pathobiology Program, Medical University of South Carolina, Charleston, SC USA; 2 Department of Pathology and Laboratory Medicine, Medical University of South Carolina, Charleston, SC USA; 3 National Institute of Standards and Technology, Chemical Sciences Division, Hollings Marine Laboratory, Charleston, SC USA; 4 Department of Biological Sciences, University of South Carolina, Columbia, SC USA; 5 Department of Biological Sciences, Salisbury University, Salisbury, MD USA; 6 Department of Pediatrics and Neonatology, Medical University of South Carolina, Charleston, SC USA

## Abstract

Evidence indicates that obesity can be promoted by chemical ‘obesogens’ that drive adiposity, hunger, inflammation and suppress metabolism. Dioctyl sodium sulfosuccinate (DOSS), a lipid emulsifier and candidate obesogen *in vitro*, is widely used in processed foods, cosmetics and as stool softener medicines commonly used during pregnancy. *In vivo* testing of DOSS was performed in a developmental origins of adult obesity model. Pregnant mice were orally administered vehicle control or DOSS at times and doses comparable to stool softener use during human pregnancy. All weaned offspring consumed only standard diet. Adult male but not female offspring of DOSS-treated dams showed significantly increased body mass, overall and visceral fat masses, and decreased bone area. They exhibited significant decreases in plasma adiponectin and increases in leptin, glucose intolerance and hyperinsulinemia. Inflammatory IL-6 was elevated, as was adipose Cox2 and Nox4 gene expressions, which may be associated with promoter DNA methylation changes. Multiple significant phospholipid/sterol lipid increases paralleled profiles from long-term high-fat diet induced obesity in males. Collectively, developmental DOSS exposure leads to increased adult adiposity, inflammation, metabolic disorder and dyslipidemia in offspring fed a standard diet, suggesting that pharmaceutical and other sources of DOSS taken during human pregnancy might contribute to long-term obesity-related health concerns in offspring.

## Introduction

Obesity and diabetes are chronic metabolic diseases affecting human populations worldwide. These metabolic disorders have increased to unprecedented levels over the past several decades. In the United States, nearly 40% of adults are obese and 7.7% are morbidly obese^1^. Also, children and adolescents ages 2–19 are affected, with 18.5% obese and 5.6% morbidly obese^1^. Body mass index (BMI) rose 2.3 kg/m^2^ from 1988 to 2006^2,3^. Rates of diabetes and metabolic syndrome have also increased and are often coupled with obesity^4,5^. Caloric imbalance (greater intake/lower expenditure) and genetics are important drivers of obesity. Despite increased efforts in nutrition and fitness education, obesity rates fail to decline and are estimated to reach 51% of the US population by 2030^6^. However, increasing numbers of obesity cases cannot be attributed purely to genetics. GIANT consortium analyses indicate that only 2.7% of increases in BMI can be ascribed to genetics and that the total genetic contribution will only reach 20% once all such genes are identified^7,8^. Such “missing heritability” cases have been attributed to gene-by-environment interactions^9,10^. For example, familial heritability and obesity-discordant twins studies and complex trait analyses^11,12^ indicate that combinations of polymorphisms can account for at most 30–37% of the variance associated with BMI^13,14^. Environmental exposures, especially during development, have gained recognition as playing important roles in obesity and metabolic syndrome^15^. Developmental exposures that impact obesity include stress, diet, pollutants, pharmaceuticals and personal care products^16–19^. Chemicals that promote obesity and metabolic syndrome have been termed ‘obesogens’^20,21^. Obesogens can drive obesity by multiple molecular mechanisms. The most researched mechanism involves obesogens acting as agonists of the nuclear receptor PPARγ. Furthermore, hormone receptor assays are often utilized for the *in vitro* identification of potential obesogens, prior to *in vivo* validation^10^. Published work from our group identified the anionic surfactant, dioctyl sodium sulfosuccinate (DOSS), as a probable obesogen using *in silico* modeling, *in vitro* receptor ligand binding and transactivation assays and murine preadipocyte adipogenic differentiation^22^. DOSS (CAS #577-11-7) is also known as AOT, Aerosol OT, diethylhexyl sodium sulfosuccinate, Colace, Docusate sodium, and many other names. Structurally similar obesogens have been classified in literature as PPARγ agonists, including the anionic surfactants sodium dodecyl sulfate (SDS) and sodium dodecylbenzenesulfonate (SDBS), which suggest that surfactants/dispersants constitute a previously unidentified and unexplored class of obesogens^23^. Epidemiological studies previously identified positive correlations between chemicals measured during pregnancy (e.g. phthalates and persistent organic pollutants) and childhood obesity^24–26^. Exposures during development can profoundly impact epigenetic patterning that persistently impact later life health trajectories^27–29^. As with the other surfactant PFOA, DOSS will be evaluated for its obesogenic potential *in vivo* during development^30^. Pregnancy associated exposure may result from other possible sources of DOSS, including dietary emulsifying agents (e.g. colored/flavored powdered beverages) and personal care products^31–34^. Approximately 11–38% of pregnant women experience constipation during pregnancy^35^, and DOSS in the form of Colace/Docusate stool softener is the treatment of choice, being prescribed up to 500 mg/day^36,37^. In addition, nearly 2 million gallons of COREXIT dispersants, containing DOSS as a principle component, were used to clean up the Deepwater Horizon oil spill.

In the current study, C57BL/6 mouse dams were exposed to DOSS following a dose and exposure scenario relevant to Colace/Docusate stool softener use by pregnant women. The C57BL/6 mouse line was chosen as it is commonly used for diet-induced obesity studies. The offspring of exposed dams were examined using markers of adiposity and metabolic syndrome and a combination of morphometrics (e.g. body composition, circulating adipokines and cytokines, tissue gene expression, glucose tolerance and epigenetic marks such as DNA methylation). This study followed the precedent set by other *in vivo* studies, which evaluated obesogens that act as ligands to activate PPARγ, including the phthalate metabolite MEHP^38^, the antifouling paint tributyltin^39^, surfactant PFOA^30^, and the pesticide triflumizole^40^. Collectively, the results generated in mice demonstrate that doses of DOSS with no apparent affect on pregnancy can promote long-term health concerns associated with obesity, metabolic dysfunction and chronic inflammation, which may be relevant to humans.

## Results

### DOSS treatment in dams increases body mass, fat mass, fat percentage and reduces bone area in adult F1 male offspring

**Figure 1:**
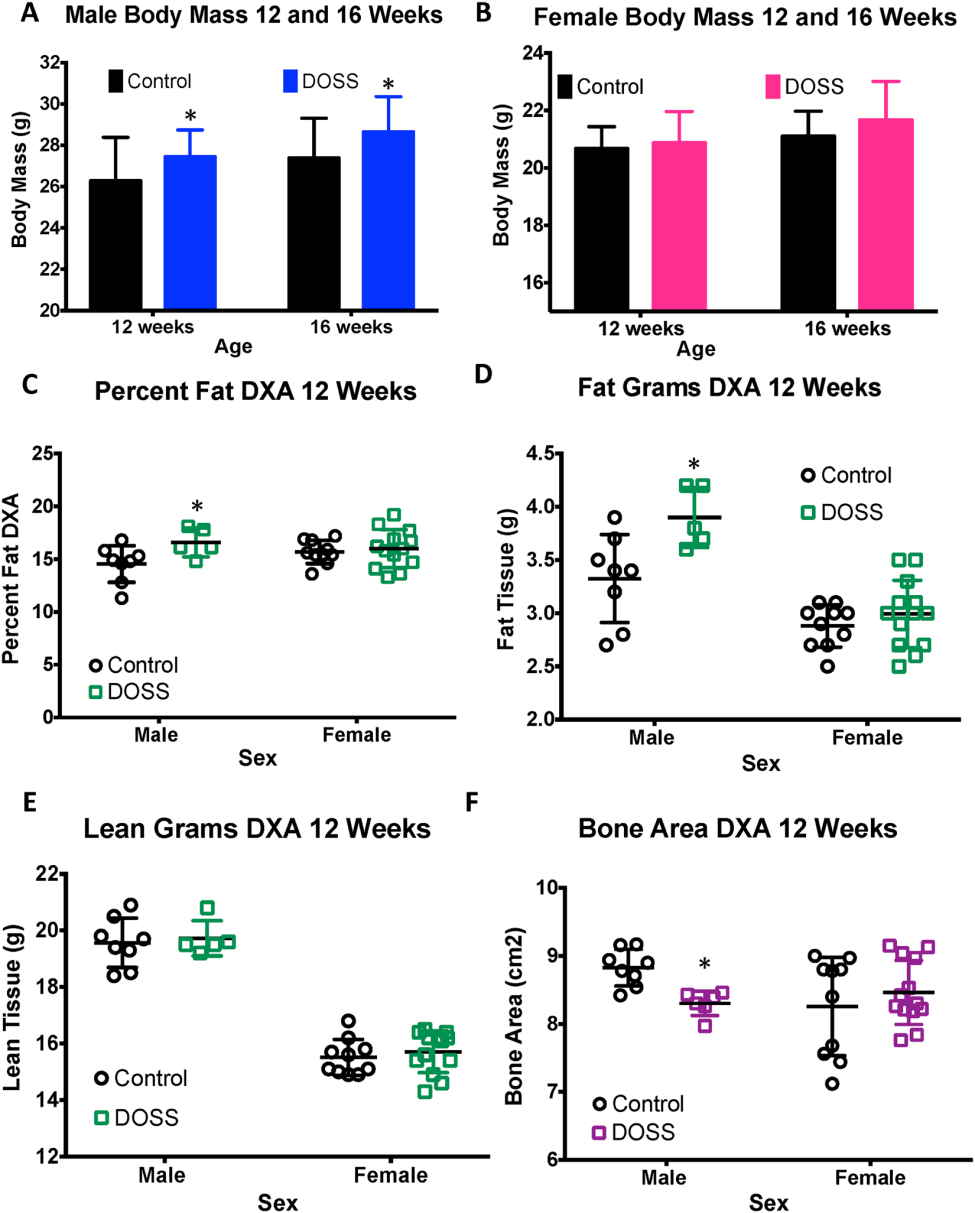
Effects of DOSS treatment on body mass and body composition in male and female F1 mice. Body mass was measured at 12 and 16 weeks of age (males, n = 28 treatment, 30 control; females, n = 16/group). At 12 weeks of age, DXA scans were used to assess body composition in male and female mice. Measurements were obtained for (**A**) male body mass, (**B**) female body mass, (**C**) percent fat, (**D**) fat mass, (**E**) lean mass, and (**F**) bone area, (**E**) bone mineral density, and (**F**) bone mineral content, (**C**) male adipose tissue mass, (**D**) female adipose tissue mass, (**E**) male adipose tissue percent, (**F**) female adipose tissue percent. Graph bars represent means and standard deviations. Male and Female mice were analyzed separately using unpaired *t*-test (*p < 0.05).

Well-established obesogens have been shown to increase overall body weight as well as adiposity or fat percentage in treated animals^21^. F1 male offspring from DOSS-treated dams were significantly heavier than control male counterparts at 12 weeks (treated mean weight: 27.4 g, control weight: 26.3 g; p = 0.02) and 16 weeks (treated mean weight: 28.6 g, control weight: 27.4 g; p = 0.01) (Fig. 1A). Although a trend of increased body mass was observed for F1 female offspring of DOSS-treated dams, no statistically significant differences in body mass were observed at either 12 or 16 weeks of age (Fig. 1B).

DXA scans were used to non-invasively assess body composition including percent body fat, fat mass, lean mass, bone area (Fig. 1C–F, bone mineral density (Supp. Fig. 1A) and bone mineral content (Supp. Fig. 1B) on a subset of animals at 12 weeks of age. Maternal DOSS treatment resulted in increased F1 male adiposity (total body fat percentage), with mean fat percentages of 16.6% and 14.5% for treatment and control F1 males, respectively (Fig. 1C, p = 0.048). There was also a statistically significant increase in fat mass for F1 male mice from treated dams with mean fat mass values of 3.9 g and 3.2 g for treatment and control F1 male mice, respectively (Fig. 1D, p = 0.020). Notably, there were no differences observed in lean mass (Fig. 1E), indicating that overall body weight gain observed was due to overall increases in fat mass. Furthermore, there was a statistically significant reduction in bone area, with 8.30 and 8.83 cm^2^ for treatment and control F1 males, respectively (Fig. 1F p = 0.001). Although results did not reach statistical significance, there was also a decrease observed in bone mineral density (BMD) and bone mineral content (BMC) for treatment vs control F1 males (Supp. Fig. 1A,B). BMC and BMD have been used as indicators of bone strength, while other studies in humans suggest bone area is a better determinant of stiffness^41,42^. Whether DOSS treatment impacts bone stiffness more than strength remains to be determined. No statistically significant differences were observed between F1 female mice from treated and control dams for DXA scan measurements (Fig. 1C–F and Supp. Fig. 1).

**Figure 2:**
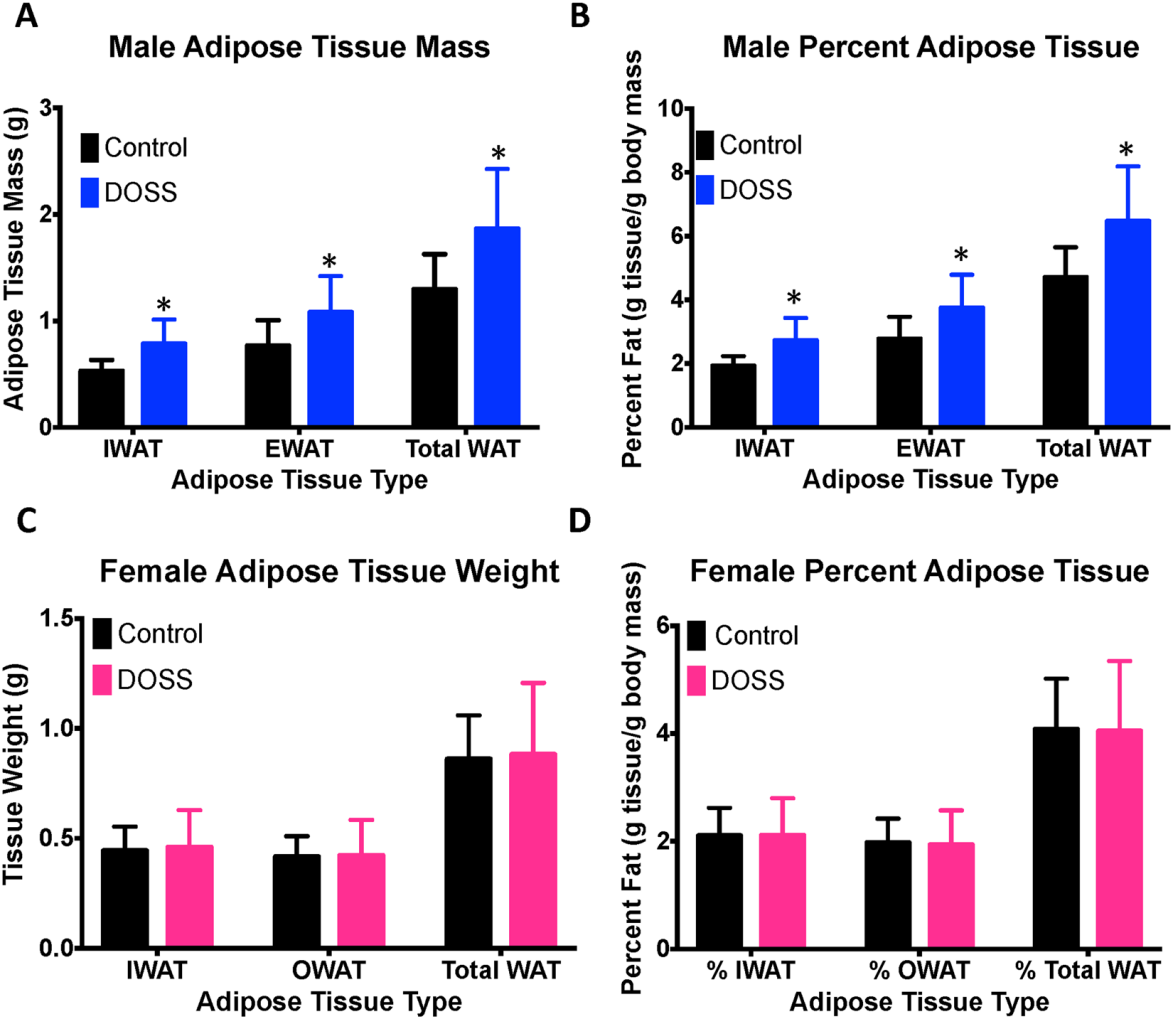
Effects of DOSS treatment on adipose tissue mass and percent adipose tissue and male and female F1 mice. At 16 weeks of age, F1 males (n = 28 treatment, 30 control) and females (n = 16/group) were sacrificed and inguinal and gonadal adipose tissue was weighed and collected. Adipose tissue percent was calculated as the weight of adipose tissue/total body mass. Measurements were obtained for (**A**) male adipose tissue mass, (**B**) male adipose tissue percent, (**C**) female adipose tissue mass, and (**D**) female adipose tissue percent. Graph bars represent standard deviations. Unpaired students *t*-test with Welch’s correction for unequal variance was used for male mice (*p < 0.05).

At 16 weeks of age, all animals were sacrificed and gross tissue masses were recorded for inguinal (IWAT) and reproductive white adipose tissue (male epididymal EWAT and female ovarian OWAT). F1 male offspring from DOSS treated dams showed statistically significant increases in adipose tissue mass for IWAT (0.78 g DOSS vs 0.52 g control; p < 0.0001), EWAT (1.15 g DOSS vs 0.76 g control p = 0.0002) and total WAT (1.93 g DOSS vs 1.28 g control p < 0.0001) (Fig. 2A). Body fat percentage as well as fat pad specific fat percentage also significantly increased in F1 treatment males (Fig. 2B). Thus, both overall body adiposity measurements by DXA scan at 12 weeks of age and IWAT/EWAT tissue mass measurements at 16 weeks of age show increased adiposity in F1 males from DOSS treated dams. No statistically significant differences were observed in F1 females from control and treated dams for adipose tissue mass or percent (Fig. 2C,D).

### DOSS treatment in dams alters circulating adipokine levels in adult F1 male offspring

In both human and mouse studies, obesity and metabolic disorders are often associated with disease-specific disruption of multiple circulating adipokines and cytokines. In particular, a decrease in adipose tissue-derived circulating adiponectin and an increase in circulating leptin are hallmarks of obesity and metabolic syndrome^43^. Furthermore, obesity can be characterized as a chronic low-grade inflammatory disease with elevations in proinflammatory cytokines^44^. Thus, to characterize the level and type of metabolic disruption observed in offspring of dams treated with DOSS, circulating plasma adipokine (adiponectin and leptin), proinflammatory cytokine (IL-6 and TNFα) and insulin levels were chosen for analysis. Multiple diet-induced obesity studies in the literature focus on male C57BL/6 mice^45,46^. Results from the current study show only the male C57BL/6 offspring of treated dams to display statistically significant DOSS-associated phenotypes (Figs 1 and 2). Therefore, only F1 male offspring from control and DOSS-treated dams were pursued for adipokine and cytokine level measurements.

**Figure 3:**
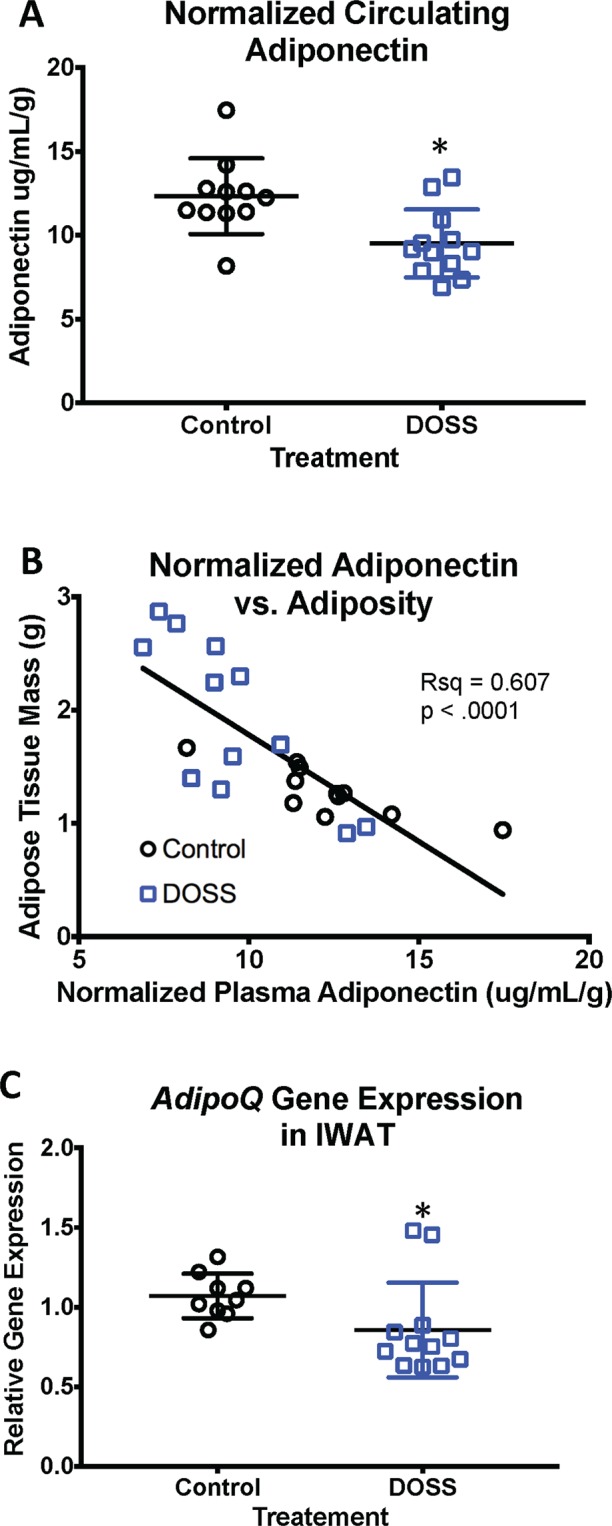
Developmental DOSS treatment alters expression and circulating levels of adiponectin in male F1 mice. At time of sacrifice (16 weeks), plasma and adipose tissue were collected (n = 12/group). Circulating levels of adiponectin were determined using MSD assays, gene expression using RNA isolation, along with cDNA conversion and qPCR using the delta delta Ct method with *Hprt* as the housekeeping gene. Results are shown for (**A**) normalized circulating levels of adiponectin, (**B**) normalized adiponectin correlated with fat mass, and (**C**) *AdipoQ* gene expression in IWAT. Some data points were excluded via outlier testing. Graph bars represent means and standard deviations. Linear regression was used to determine correlations between adiponectin and fat mass (*p < 0.05 unpaired *t*-test).

Adiponectin, a signaling molecule exclusively produced by adipose tissue, plays roles in insulin sensitization and glucose homeostasis^47^. To account for increases in adiposity in treated animals (Figs 1 and 2) and to be consistent with other published study methods, adiponectin levels were normalized to weight of adipose tissue (IWAT + EWAT) for all individuals^48^. The normalization of adiponectin level compared to adiposity is a means to measure rate change. Relative circulating adiponectin levels (microgram adiponectin/mL plasma*gram adipose tissue) were significantly lower in F1 male offspring from DOSS-treated dams (9.51 μg/mL*g) than in males from control dams (13.30 μg/mL*g), with a p = 0.003 (Fig. 3A). The data in Fig. 3B demonstrates that DOSS-associated adiposity results in less production of circulating adiponectin.

The statistically significant reduction in normalized adiponectin levels observed in treated animals could be due to significant changes in white adipose tissue depots expressing *AdipoQ*, the gene encoding adiponectin (Supp. Table 1). Consistent with this, a statistically significant down regulation of *AdipoQ* expression (p = 0.015) was observed in F1 male offspring IWAT tissues from treated versus control dams (Fig. 3C). Although not statistically significant, decreased gene expression of *AdipoQ* was observed in male gonadal (EWAT) adipose tissue as well (data not shown). These data further support the hypothesis that DOSS exposure during pregnancy results in decreased adiponectin in adult male offspring at 16 weeks. A non-statistically significant trend for lower *AdipoQ* expression was observed in adult female offspring IWAT of treated dams (data not shown). These results suggest that no change in circulating adipokine levels in females would be observed, further supporting the association between DOSS exposure, adipokine levels and overweight phenotype in male, but not female animals.

**Figure 4:**
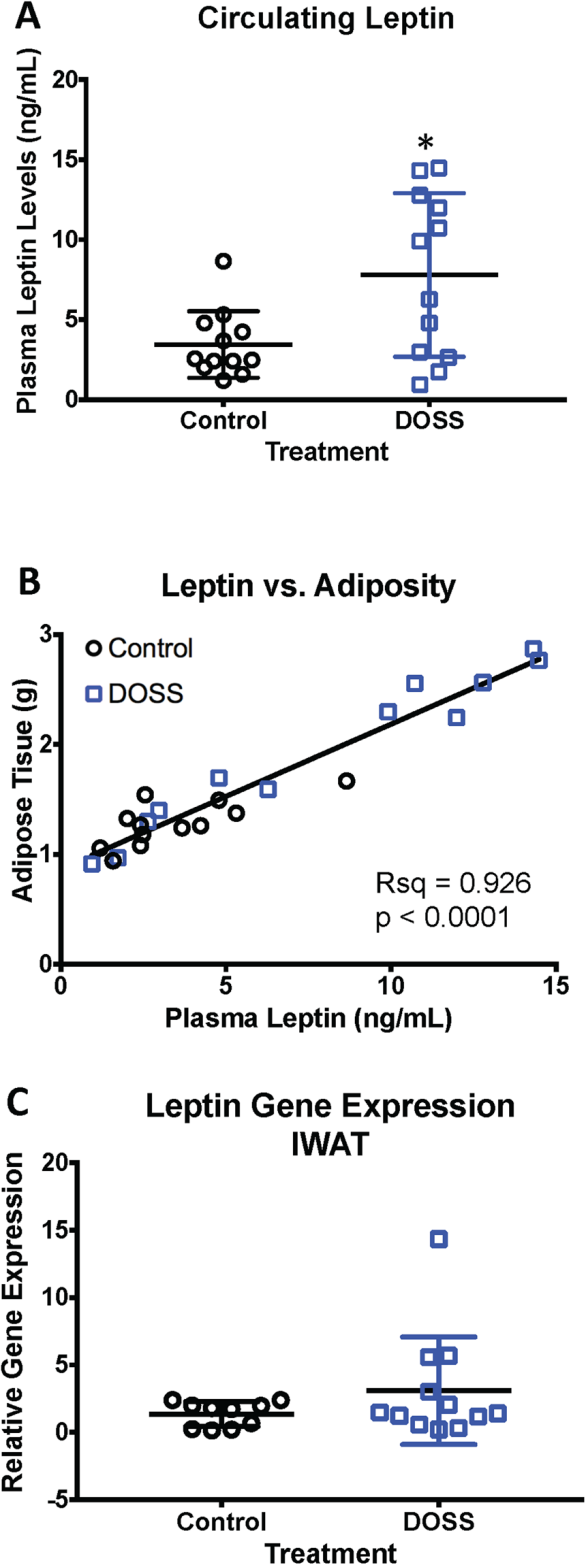
Developmental DOSS treatment alters circulating levels of leptin in male F1 mice. At time of sacrifice (16 weeks), plasma and adipose tissue was collected (n = 12/group). Circulating levels of leptin were determined using MSD assays. Gene expression was determined via RNA isolation, cDNA conversion and qPCR using the delta delta Ct method with *Hprt* as the housekeeping gene. Results are shown for (**A**) normalized circulating levels of leptin, (**B**) leptin correlated with fat mass, and (**C**) *leptin* gene expression in IWAT (n = 12/group). Graph bars represent means and standard deviations. Linear regression was used to determine correlations between adiponectin and leptin and fat mass (*p < 0.05 unpaired *t*-test).

Leptin, an adipokine that regulates satiety and appetite, has been shown to be positively correlated with adiposity and obesity^49^. While other fat depots are capable of producing and secreting more leptin, models based on circulating leptin levels are likely more reliable than IWAT gene expression for whole body response to satiety and appetite. Therefore, this study provided circulating leptin levels. Significantly higher levels of circulating leptin (p = 0.033) were observed in adult F1 males from DOSS-treated dams compared to controls (Fig. 4A). A positive relationship between leptin and adiposity markers is well documented in both humans and mice^50,51^. Our work demonstrates this same trend as circulating leptin levels were positively correlated with fat mass for DOSS treatment (Fig. 4B). Although some treatment F1 males displayed higher expression of the leptin gene in IWAT relative to controls, the differences were not statistically significant (Fig. 4C).

### DOSS treatment in dams produces a persistent inflammatory state in adult F1 male offspring

**Figure 5:**
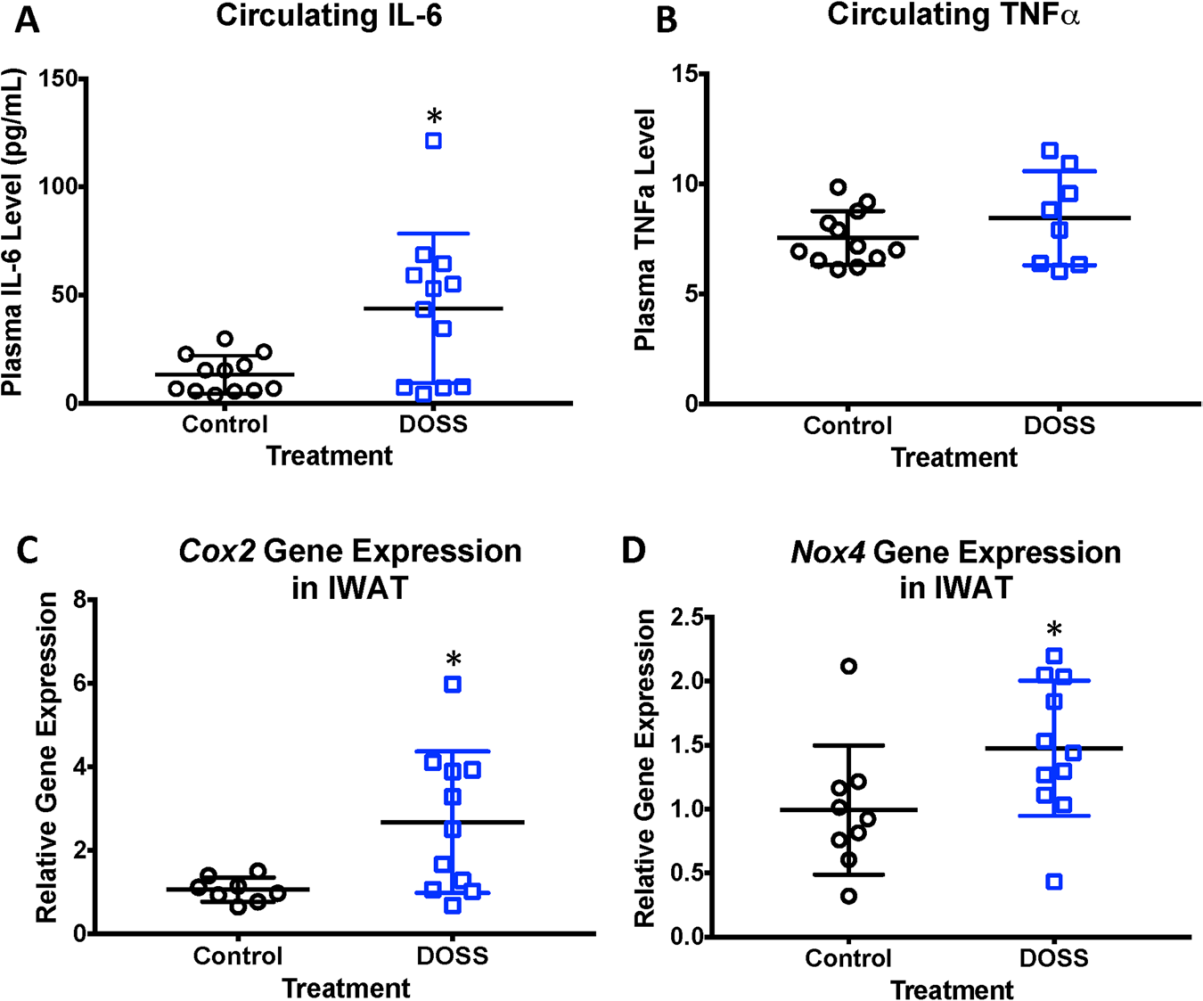
Developmental DOSS treatment promotes a proinflammatory state in adult F1 male mice. At time of sacrifice (16 weeks), plasma and adipose tissue were collected (n = 12/group). Circulating levels of IL-6 and TNF-α were determined using MSD assays, gene expression using RNA isolation, along with cDNA conversion and qPCR using the delta delta Ct method with *Hprt* as the housekeeping gene. Results are shown for (**A**) circulating levels of IL-6 in plasma, (**B**) circulating levels of TNF-α in plasma, (**C**) Cox2 gene expression in IWAT tissue, and (**D**) Nox4 gene expression in IWAT tissue (*p < 0.05 unpaired t-test). Some data points were excluded via outlier testing. Graph bars represent means and standard deviations.

Obesity and type 2 diabetes (T2D) are often referred to as a chronic inflammatory diseases of adipose tissues, which are presumably driven by infiltration of adipose tissue with macrophages^52^. IL-6 is a proinflammatory cytokine secreted by immune cells. Circulating IL-6 levels were measured to determine if offspring of DOSS-treated animals showed indications of such an inflammatory state. Significantly higher circulating levels of IL-6 in treatment (48.12 pg/mL) versus control (13.70 pg/mL) adult F1 males were observed at p = 0.011 (Fig. 5A). Unlike adiponectin and leptin, a correlation between adipose tissue weight and circulating IL-6 levels was not observed in either treatment or control animals (Supp. Fig. 2A). IL-6 gene expression changes were also monitored in IWAT adipose tissue for potential associations with inflammation. No statistically significant differences in IL-6 gene expression were observed between treatment and control animals (Supp. Fig. 2B) despite increased circulating levels of IL-6 in treatment animals (Fig. 5A). This finding may suggest the contribution of other immune cell/fat depots to overall circulating IL-6 levels. Within adipose tissue, the stromal vascular fraction accounts for 90% of the IL-6 expression in adipose tissue even though it represents a small percentage of adipose tissue cells^53^.

TNFα is another inflammatory cytokine secreted by immune cells and has been shown to be positively correlated with obesity and T2D. Therefore, mouse plasma were analyzed for levels of TNFα. The mean circulating TNFα was higher in treatment adult F1 males (8.44 pg/mL) compared to controls (7.54 pg/mL), although not statistically significant (Fig. 5B).

Other genes investigated included cyclooxygenase 2 (Cox2) and NADPH oxidase (Nox4; Supp. Table 1). Elevated expression of Cox2 and Nox4 are associated with the presence of reactive oxygen species and systemic chronic inflammation^54,55^. Both Cox2 and Nox4 expressions were significantly increased in IWAT for DOSS-treatment versus control adult F1 males (Fig. 5C,D). Thus, the increased expression of multiple inflammatory markers in this work suggests that a state of chronic inflammation exists in offspring of DOSS-treated dams.

### DOSS treatment in dams produce altered DNA promoter methylation of IL-6 and Cox2 genes in adult F1 male offspring

Increased levels of gene expression (Cox2 and Nox4) and circulating levels of IL-6, adipokines and cytokines were observed in 16 week-old F1 males of DOSS treated dams. These findings suggest alterations of promoter methylation at these loci in IWAT. Therefore, promoter methylation was investigated at these loci, as well as at previously identified gene promoters reported in the literature to be regulated by DNA methylation in response to high-fat diet, obesity or T2D (*Leptin*, *Pparg*, *Glut4*, *Fasn*, *Irs1*, *Hmox1*, *Fabp4*). A complete list of loci, bisulfite primers and references used in this work are presented in Supp. Table 2.

The promoter region of the IL*-*6 gene in IWAT tissue was investigated for changes in DNA methylation. Five previously identified CpG sites roughly 300 bp upstream of the IL-6 transcription start site were evaluated for methylation status^56^. Results in Supp. Fig. 3A show DOSS treatment to be associated with significantly reduced methylation specifically at CpG site 2 by two-way ANOVA (p = 0.004) and by Sidak’s post hoc test (p = 0.045). Although there was no statistically significant correlation (p = 0.705) between IWAT gene expression and DNA methylation at CpG site 2 (Supp. Fig. 4A), there was a statistically significant inverse correlation (p = 0.011) between IL-6 CpG site 2 methylation and circulating IL-6 levels. (Supp. Fig. 3B). To the best of our knowledge this is the first significant finding of IL-6 CpG site 2 methylation status in adipose tissue as it relates to circulating IL-6 levels, though it’s biological importance remains to be determined. Although multiple tissue sources of IL-6 contribute to circulating levels, the potential of CpG site 2 to serve as a biomarker for the persistent effects in offspring of DOSS exposed mothers warrants further investigation.

Changes in Cox2 promoter hypermethylation upon exposure have been linked to Cox2 gene expression in other studies^57^. Therefore, fourteen CpG sites approximately 500 bp upstream of the *Cox2* transcription start site were evaluated for methylation status. Results demonstrate that although DOSS treatment was not significantly associated (p = 0.128) with *Cox2* promoter methylation in this region (Supp. Fig. 3C), average DNA methylation at CpG site 1 was significantly reduced (p = 0.005) in treatment males (Supp. Fig. 3D). However, there was only a modest correlation (p = 0.280) between CpG site 1 methylation and *Cox2* gene expression (Supp. Fig. 4B).

Bisulfite sequencing for six CpG sites in the adiponectin promoter were targeted (i.e. four adiponectin region 2 sites, associated with high-fat diet induced gene expression, and two nearby adiponectin region 1 sites having no previously documented associations with gene expression). Statistically significant hypomethylation was observed in region 1, particularly at CpG site 1 (Supp. Fig. 4C) in tissues from treatment males. There was no statistically significant difference in region 2 promoter methylation between treatment and controls. Decreased levels of circulating adiponectin and gene expression in IWAT tissue suggest the possible hypermethylation of the adiponectin promoter at each CpG site in region 2, resulting in repression of gene expression upon DOSS treatment (Supp. Fig. 4D). Region 2 CpG site 3 showed that 58% of DOSS treatment animals had over 80% methylation compared to 29% of controls with over 80% methylation (Supp. Fig. 4E). However, no statistically significant correlations were observed between region 2 CpG site 3 methylation and gene expression (Supp. Fig. 4E). Furthermore, no statistically significant differences in promoter methylation were observed for *Leptin*, *Fasn*, *Pparg*, *Glut4*, *Irs1*, *Fabp4*, or *Hmox1* (Supp. Table 3).

### DOSS treatment in dams induces glucose intolerance in adult F1 male offspring

**Figure 6:**
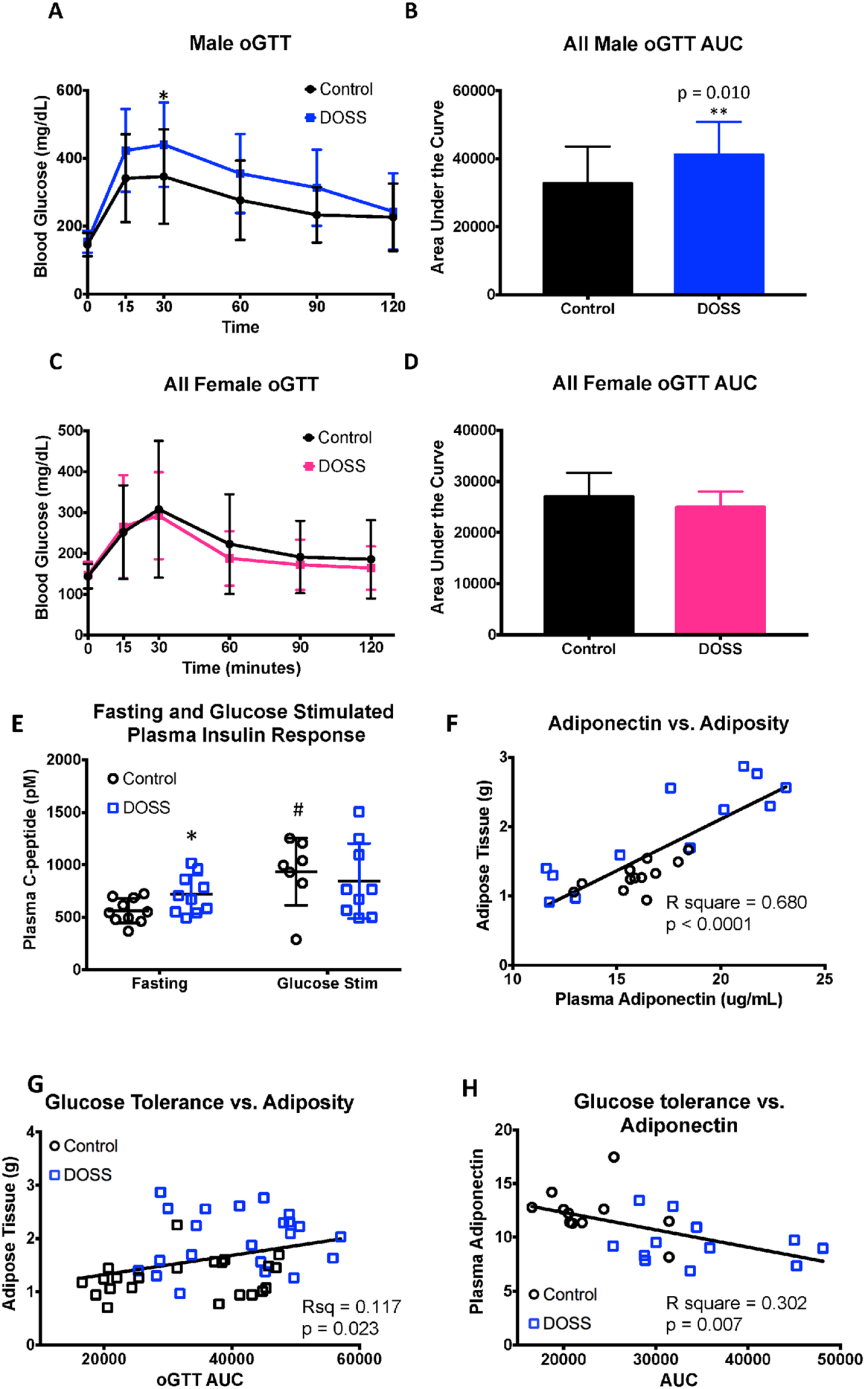
Developmental DOSS treatment in dams promotes glucose intolerance, and alters expression and circulating levels of insulin in adult F1 male offspring. Pregnant C57BL/6J dams were treated with either vehicle control or DOSS from E11.5 through weaning. F1 pups were assessed for indications of metabolic syndrome at 12 weeks using oral glucose tolerance testing. Basal glucose measurements were taken after fasting for 6 hours. Mice were then administered a 2 mg/g bolus of glucose and blood glucose was measured at 15, 30, 60 and 120 minutes. Blood glucose measurements for all male (n = 22/group) mice are shown in (**A**) with corresponding area under the curve values in (**B**). Blood glucose measurements for all female (n = 16/group) mice are shown in (**C**). Blood glucose area under the curve values for females are shown in (**D**). Two-Way Anova was used to determine significant differences in blood glucose at different time points (*p < 0.05). Area under the curve values were calculated for each mouse, pooled and student’s *t*-tests were used to determine significance (**p < 0.01). Plasma and adipose tissue were collected at time of sacrifice. Circulating levels of C-peptide were determined using Alpco ELISA assays. Linear regression was used to determine correlations between adiponectin, adipose weight and glucose tolerance. Results are shown for (**E**) fasting and glucose stimulated C-peptide levels at 12weeks, (**F**) raw adiponectin correlated with fat mass, (**G**) adipose tissue weight correlated with oGTT AUC as a measure of glucose tolerance, (**H**) normalized adiponectin correlated with oGTT AUC as a measure of glucose tolerance (*p < 0.05 unpaired *t*-test, control vs. DOSS treatment; #p < 0.05 unpaired *t*-test, fasting vs. glucose stimulated C peptide). Graph bars represent means and standard deviations.

Glucose tolerance tests are used clinically to assess metabolic function in humans at risk for diabetes and experimentally in obesity and diabetic mouse models. To determine the effect of maternal DOSS treatment during development on glucose tolerance in adult offspring, oral glucose tolerance tests were performed at 12 weeks of age on offspring of control and DOSS-treated dams. Results demonstrate that only male offspring of DOSS treated dams exhibit marked glucose intolerance (Fig. 6). Specifically, Fig. 6A shows statistically significant higher blood glucose levels at 30 minutes in male offspring of DOSS treated vs. control dams. Similarly, these DOSS treatment F1 males show a significantly higher overall glucose tolerance impairment (p = 0.010), as indicated by the area under the curve (AUC) for the entire time course (Fig. 6B). No specific time points or AUC differences were observed amongst the female offspring (Fig. 6C,D). Male-specific results are commonly observed in C57BL/6 mouse models for high-fat diet induced obesity^58,59^.

C-reactive peptide (C-peptide), a more stable marker for insulin due to its longer half-life, is released in a 1:1 ratio to insulin, making it more conducive for plasma studies with variable storage times and conditions. Therefore, circulating C-peptide levels were measured as a stable surrogate for insulin secretion in male offspring (n = 10/group) to determine whether glucose intolerance in DOSS treatment F1 males resulted from insulin desensitization marked by hyperinsulinemia. Significantly higher levels of fasting C-peptide (p = 0.040) was measured in male offspring of DOSS-treated dams (718 pM) relative to controls (563 pM; Fig. 6E). Furthermore, upon glucose stimulation, a statistically significant increase (p = 0.020) in C-peptide levels was observed only in control animals (Fig. 6E). These results suggest that adult male offspring of DOSS-treated dams demonstrate hyperinsulinemia and blunted insulin responsiveness to glucose stimulation. Primarily glucose intolerance and adiponectin, but also obesity and adiponectin have been described to have an inverse relationship^60^, while positive correlations between circulating adiponectin and adipose fat mass have been observed^61,62^. Consistent with these results, raw adiponectin level and glucose intolerance are positively correlated with adipose tissue mass in our animals (Fig. 6F,G), and adiponectin levels are negatively correlated with glucose intolerance (Fig. 6H).

### DOSS treatment in dams produces adult F1 male offspring with elevated circulating phospholipid patterns commonly observed in long-term high-fat diet induced obesity and diabetes

**Figure 7:**
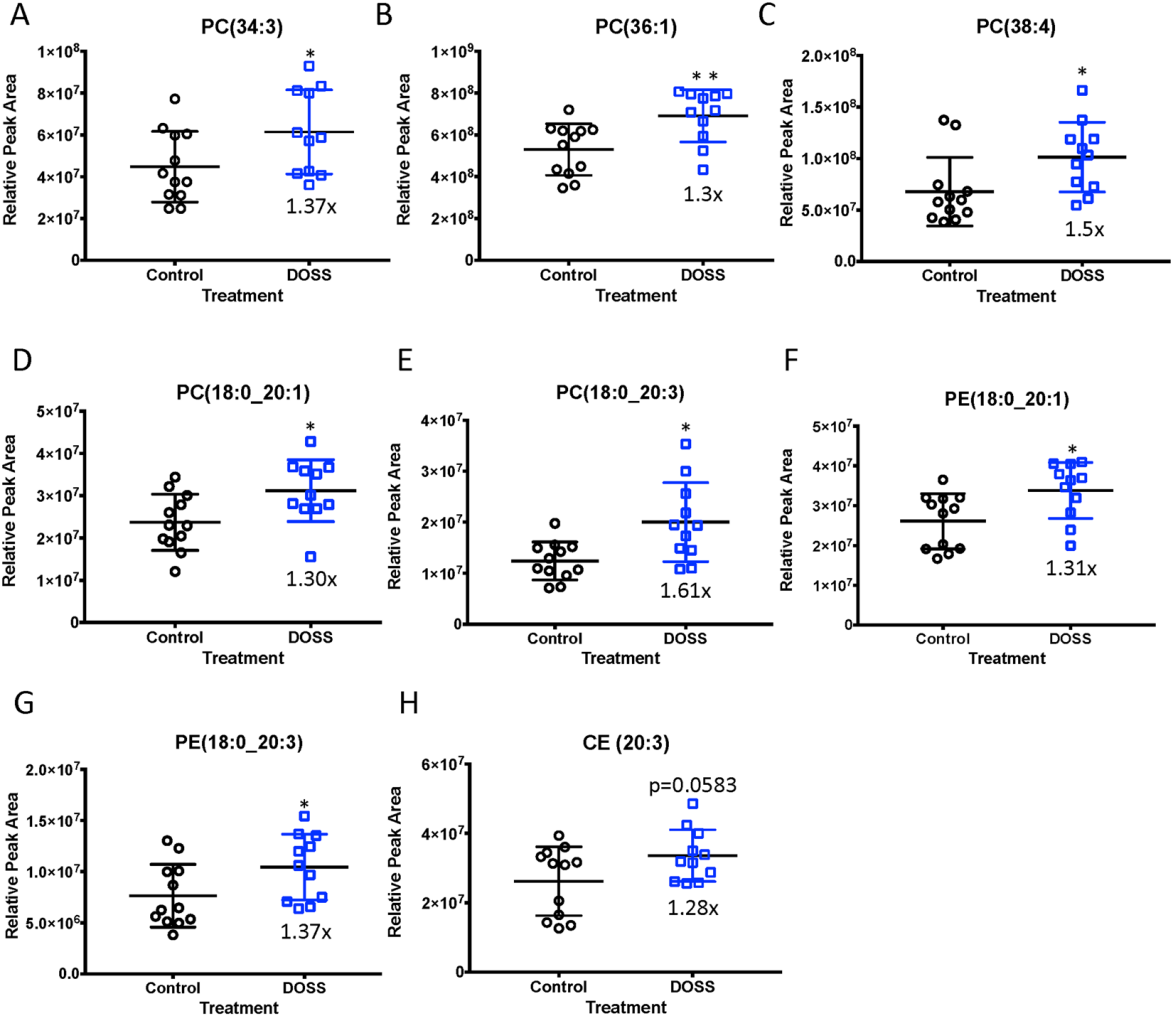
DOSS treatment in dams promotes persistent changes in circulating phospholipids in adult F1 male offspring. At time of sacrifice plasma was collected from F1 animals (n = 12/group). Untargeted lipidomics was used to determine the presence of circulating lipids. Lipid Match was used to identify lipid species based on unique m/z ratios and retention times. Relative peak area was calculated in FRAMe. Relative peak areas between DOSS treated and vehicle control F1 males are shown for (**A**) phosphatidyl choline PC(34:3), (**B**) PC(36:1), (**C**) PC(38:4), (**D**) PC(18:0_20:1), (**E**) PC(18:0_20:3), (**F**) phosphatidylethanolamine PE(18:0_20:1), (**G**) PE(18:0_20:3), and (**H**) cholesterol ester CE(20:3) (*p < 0.05 **p < 0.01 unpaired *t*-test control vs. DOSS treatment). Some data points were excluded via outlier testing. Graph bars represent means and standard deviations.

Due to recent advancements in mass spectrometry (MS) and ultra-high performance liquid chromatography (UHPLC) untargeted lipidomic applications have broadened to investigate lipid profiles associated with obesity and diabetes^63–65^. Biomarkers identified using this approach have the potential to elucidate cellular mechanisms associated with disease progression. Therefore, an untargeted lipidomics workflow was incorporated to identify significant and persistent changes in plasma lipid profiles as a result of DOSS exposure during development. Overall, 664 features were identified in positive MS scan mode and 171 features identified in negative MS scan mode. Various phospholipids (e.g. phosphatidyl choline (PC) and phosphatidylethanolamine (PE)) and sterol lipid (e.g. cholesterol ester (CE)) species were increased in 16 week-old F1 male offspring of DOSS-treated dams. As shown in Fig. 7A–H, the following lipid species demonstrated statistically significant or near significant increases in relative peak areas: PC(34:3) (p = 0.043), PC(36:1) (p = 0.005), PC(38:4) (p = 0.012), PC(18:0_20:1) (p = 0.018), PC(18:0_20:3) (p = 0.038), PE(18:0_20:1) (p = 0.015), PE(18:0_20:3) (p = 0.044), and CE(20:3) (p = 0.058). Several of the lipid species annotated in this work have also been highlighted in literature^66^ as putative biomarkers for high-fat diet induced obesity in C57BL/6 mice, including PC(36:1), PC(38:3), PC(38:4) and CE(20:3). Lysophosphatidylcholine (LPC) lipid species have been shown to be reduced in response to high-fat diet, obesity and diabetes^67^. Reductions in several novel LPCs were observed in response to DOSS exposure during development, including LPC 15:0, 17:0, 19:0, 20:0, 22:0, 22:1 and 24:1. These results suggest that developmental exposure to DOSS followed by a normal *ad lib* diet promotes persistent dyslipidemia akin to long-term high fat-diet induced obesity in adult mice.

## Discussion

This work provides evidence in support of the hypothesis that DOSS acts as an obesogen *in vivo* via metabolic disruption. Exposure to DOSS during development resulted in impaired glucose tolerance, insulin desensitization, increases in adiposity, and altered gene expression, circulating levels of adipokines, cytokines and phospholipids. These changes, observed only in the male mice studied, are consistent with obese, diabetic and metabolic syndrome phenotypes demonstrated in literature. Previous studies have shown C57BL/6 mice to demonstrate sexually dimorphic metabolic and obesogenic effects. For instance, female mice fed a high-fat diet for 14 weeks were protected from metabolic syndrome, unlike the male counterpart^68,69^. In a prenatal exposure model for MEHP in C57BL/6 mice, markers of obesity were observed in male offspring but not in female offspring^38^. The recently identified metabolic disruptor, tolyfluanid, similarly exerted a metabolic disrupting phenotype only in adult male C57BL/6 mice. The neuroendocrine axis (cortisol, HPA stimulation) may differ also by sex and is worth considering as a potential modulator of these effects. Conversely, C57BL/6 mice exposed during development to the obesogen, TBT, showed the obesogenic phenotype in both the female and male offspring^39^. Triflumazole, a PPARγ agonist induced obesogenic effects including MSC fate commitment only in male mice of the CD1 background^40^. Multiple direct or indirect modes of action could be conjectured to be responsible for the gender-specific effects of DOSS on offspring in the model presented. For example, direct effects could occur via PPAR signaling and sexually dimorphic expression of PPARγ and protein partners or co-factors^70,71^. Downstream or indirect effects could be mediated via the placenta. For example, in spiney mice (*Acomys cahirinus*) the placentas associated with male and female fetuses are structurally dimorphic^72,73^. In C57BL/6 mice specifically, imprinted gene expression and DNA methylation were found to be sexually dimorphic in placentas under a high-fat diet^74,75^.

Results demonstrate that DOSS exposure during development induced glucose intolerance and increased adiposity, coupled with a reduction in adiponectin gene expression and circulating levels. This inverse relationship between glucose intolerance/increased adiposity and reduced adiponectin is well documented in the literature as a marker of metabolic syndrome in humans and mice^76^. Because adiponectin signaling is involved in insulin sensitization, the observed reduction in adiponectin in this work may be a reason for the observed glucose intolerance. Literature has shown that adiponectin treatment ameliorates hyperglycemia symptoms in T2D patients and accentuate effects in adiponectin knockout mice on a high fat-diet^77^. Human cohort studies have identified lower circulating levels of adiponectin in T2D individuals compared to controls. In a study of obese and non-obese T2D individuals, a positive correlation between BMI and serum adiponectin was observed and all T2D individuals showed lower adiponectin levels than the control group^78^. Given that, in F1 DOSS treatment mice, normalized adiponectin levels were more consistently lower compared to leptin level increases (whose consistency were dependent on a two-gram adipose tissue threshold; Fig. 4B), adiponectin may serve as a better marker than leptin for predicting developmental DOSS exposure and for predicting which individuals exposed to DOSS are likely to develop metabolic syndrome.

Hyperinsulinemia and blunted insulin responsiveness, measured in this work using fasting C-peptide levels, are commonly associated with impaired glucose tolerance, increased adiposity, and reduced adiponectin levels. F1 male offspring of DOSS-treated dams demonstrated all of these trends that have been recognized in literature as indicators of metabolic syndrome^79^. The observed impaired glucose tolerance and elevated blood glucose could also be a result of malfunctioning glucose transporters like Glut4 in skeletal muscle tissue, the tissue that first responds to insulin stimulation^80,81^.

Although no significant increase in DNA promoter methylation for adiponectin was observed, gene expression decreased in F1 male offspring of DOSS-treated dams. Literature has shown that a high-fat diet can induce hypermethylation in region 2 of the adiponectin promoter and that this correlated with decreased adiponectin gene expression^82^. Future studies could investigate other adiponectin promoter regions for regulatory CpG sites. For instance, 500 bp upstream of the region investigated in this study, there is an *Ebf1* transcription factor binding site and *Ebf1* binding has been shown to be reduced at methylated sites^83^.

Chronic low-grade inflammation, especially in adipose tissue, is another hallmark of obesity and metabolic syndrome. In this study, treated mice had higher circulating levels of IL-6, a cytokine commonly found to be positively correlated with diabetes and obesity^84^. Although an increase in IL-6 gene expression in IWAT was not observed, there was a decrease in promoter methylation at a single CpG site which was negatively correlated with circulating IL-6 levels, suggesting a possible functional role for DNA methylation in the IL-6 promoter. Given that adipose tissue is comprised of multiple cell types (including adipocytes, mesenchymal stem cells, immune macrophage and lymphocytes), and that cells predominantly expressing IL-6 (such as macrophage and preadipocytes) likely constitute a small proportion of the total cell population, future studies should separate the stromal vascular fraction from adipoctytes and perform gene expression and DNA methylation in both cell fractions to gain a more comprehensive understanding of IL-6 regulation. IL-6 promoter methylation in adipose tissue has not been investigated with regards to obesity, but in humans IL-6 promoter methylation has been investigated in circulating white blood cells with differing results. In circulating white blood cells Kirchner *et al*. observed obesity associated hypomethylation of the IL-6 promoter with no correlation to circulating levels^85^ while Kyung Na *et al*. (2015) observed obesity associated hypermethylation of the IL-6 promoter^86^ without having determined circulating levels. Determining if white blood cell IL-6 methylation correlates with adipose tissue IL-6 methylation would strengthen the results observed in human studies.

Increases in expression of two other genes commonly associated with a systemic inflammatory state and obesity, Cox2 and Nox4, were observed in IWAT tissues of F1 males from DOSS treated dams^54,55^. Cox2 has been shown to be an essential mediator in obesity-induced adipose tissue inflammation and insulin resistance as it is an enzyme responsible for the production of arachidonic acid-derived proinflammatory eicosanoids^87^. Eicosanoid profiling focusing on products of Cox2 mediated reactions in adipose tissue or plasma from males developmentally exposed to DOSS would further elucidate the role of DOSS induced inflammation *in vivo*. Others have identified increased Cox2 expression in IWAT in response to maternal obesogen exposure, specifically PAHs^88^. In F1 males from DOSS treated dams from this study, upregulated transcription of Cox2 and hypomethylation of one CpG site in the promoter region of Cox2 in IWAT was observed. This site was the most upstream site interrogated and was more methylated than the other sites examined (Fig. 5C, Supp. Fig. 3C; sites 2–14). It is possible that the regulatory region associated with DOSS-mediated upregulation of Cox2 in adipose tissue may be located further upstream given the trend observed at CpG site 1. While Cox2 methylation has been investigated in other tissue types and plays a role in progression of some cancers^87^, to the best of our knowledge this is the first investigation into adipose tissue Cox2 methylation as it relates to obesity and diabetes.

Given that indicators of low-grade inflammation were observed at the epigenetic (IL-6 and Cox2 hypomethylation), gene expression (*Cox2* and *Nox4*) and protein levels (circulating IL-6), inflammation is likely to be a key mechanism by which developmental DOSS exposure could promote adult onset metabolic syndrome. Additionally, since we observed DNA methylation changes correlated with circulating levels of IL6 but observed no methylation changes in the adiponectin promoter, this may suggest a mechanism by which DOSS alters glucose metabolism. Our data suggest a possible predisposition to a proinflammatory state via hypomethylation of the IL-6 promoter, which in turn could reduce adiponectin expression and circulating levels leading to impaired glucose metabolism because *in vitro*, IL-6 and other proinflammatory cytokine treatments of adipocytes can reduce adiponectin secretion^89,90^. While, Cox2 activity is associated with a specific category of lipid mediators in inflammation, the broader field of lipidomics has emerged as a useful tool in identifying pathological pathways of diseases, including diabetes and obesity as well as to serve in identifying biomarkers of disease states^91^.

Using an untargeted lipidomics approach for the analysis of plasma samples from F1 male offspring of DOSS exposed and unexposed dams, significant profile differences in circulating PC, PE, CE, and LPC lipid species were observed (Fig. 6). There is a limited number of untargeted lipidomics studies in the literature that monitor obesogenic exposures. Van Ginneken and co-workers reported increased levels of CE(20:3) and PC(36:1) in animals fed a high-fat diet^92^. A large cohort study reported PC(36:1) as a biomarker for T2D^93^. Both lipid species, PC(36:1) and CE(20:3), were significantly increased in male offspring from DOSS-treated dams in this work. Literature has reported improved glucose tolerance in *db/db* mice incorporated in obesity and T2D studies upon treatment with PC(18:0_18:1). Eisinger and co-workers observed increases in CE(20:3), PC(38:3) and PC(38:4)^66^ all of which were significantly increased in male offspring from DOSS-treated dams in this work. In a human cohort study, PC(38:3) was associated with an increased risk in diabetes^93^. Barber and co-workers observed increases in PE(38:0), PE(36:1) and PE(38:4)^67^. Male offspring from DOSS-treated dams showed increased levels of other PE species, PE (18:0_20:1) and PE (18:0_20:3). A decrease in several LPC species from DOSS-treatment males was observed in this work, which have been negatively correlated in the literature with diet induced obesity and T2D in mice and humans^66,67,94^. Together these data suggest that developmental DOSS exposure induces phospholipid changes similar to diet induced obesity models with some distinct differences.

While this work highlights perturbed glucose and lipid metabolism resulting from developmental DOSS-treatment, alternative mechanisms not explored here may also contribute to obesogenic effects. DOSS has been shown to act as a ligand activator of PPARγ^22^, however DOSS is also likely to alter fundamental properties of lipid bilayers associated with cell membranes and organelles and molecular trafficking, given its molecular structure and function^95^. Additionally, DOSS may alter the microbiome of pregnant dams, thereby influencing maternal nutrition and transfer to pups. In humans, it has been documented that the maternal microbiome composition and maternal nutrition impact the onset of obesity and diabetes in offspring^96^. Furthermore, other food additives and emulsifiers functionally similar to DOSS have been evaluated for their impact on the microbiome and subsequent adverse health effects, including obesity and inflammatory bowel disease^97,98^. In cows, DOSS treatment has been used to eliminate rumen ciliate protozoa^99^, but was later shown to alter microbial communities, increase circulating blood glucose and alter the fatty acid composition of their milk^100^.

As EDCs have multiple mechanisms of action, it is likely that DOSS could simultaneously act as a PPARγ agonist as well as through other mechanisms not yet to explored. For example, the timing of PPARγ activation can differentially impact insulin levels. Adult exposure to strong, highly specific PPARγ agonists such as troglitazone compounds can decrease insulin levels^101^. However exposure to PPARγ agonists during development can increase the number of fat cells produced during development, leading to an obese phenotype and development of metabolic syndrome in adults, marked by hyperinsulinemia, long after exposure to the PPARγ agonist has ceased^40,102^. While protocols for measuring DOSS exist for seawater and fish tissue, they are not directly transferable to human biological samples due to differences in matrix composition and high limits of detection^103,104^. A standard protocol for measuring DOSS in biological samples (e.g. blood and breast milk) is currently nonexistent making overall human exposure difficult to assess. Facile protocols for assessing DOSS exposure and results provided herein could significantly aid in understanding the contribution of DOSS exposure during pregnancy to child metabolism, obesity and overall health. Several studies have highlighted the ineffectiveness of Colace to treat constipation, thus suggesting an opportunity to reduce prescription or eliminate use of this medication during pregnancy^105,106^.

In summary, this work provides evidence that DOSS acts as a developmental obesogen *in vivo*. This study highlights the need to develop effective DOSS detection methods for human biological samples and to further investigate the health effects associated with DOSS exposure in human populations. Upon DOSS treatment, results from male offspring varied in magnitude in that some animals exhibited a more exaggerated fat accumulation effect and others demonstrated higher/lower adiposity levels, including circulating leptin. Natural individual differences (e.g. social ranking) may be a large contributing factor to the variance observed. Other factors that may have contributed to the variance include: differences in maternal water consumption resulting in altered effective DOSS dosage levels, differences in food consumption and physical activity of pups, or seasonality of offspring birth. Additional studies are needed to determine the effect each factor contributed to the variance. Nevertheless, given the observed metabolic disruptions in male offspring from dams treated with DOSS, the use, safety and prescription of docusate (Colace) stool softener and possible significant dietary sources of DOSS during human pregnancy should face further scrutiny.

## Materials and Methods

### Animal husbandry and treatment

C57BL/6J mice were purchased from JAX^®^ Mice (Bar Harbor, ME). All mice were housed in the Medical University of South Carolina (MUSC) Division of Laboratory Animal Resources (DLAR; approved assurance number for AAALAC, Intl. #695 is A3428-01; APHIS/USDA #925, 56-R-0001) facilities and all animal experiments were performed in accordance with relevant Institute of Animal Care and Use Committee (IACUC) guidelines and regulation standards under a full board approved protocol (2187, renewal number IACUC-2018-00322, expiration date June 25, 2020). The C57BL/6 mouse strain was chosen for its susceptibility to diet and environmental induced obesity and metabolic syndrome^58,59,107^. Animals were fed a standard chow diet of Envigo/Teklad 2918 containing 18.6% crude protein, 6.2% fat, and 44.2% carbohydrates. Sterile corncob bedding was kept in cages, except when mice were fasted for oral glucose tolerance testing. Room temperature was maintained between 68–74 °F and relative humidity between 30–70%. Light was controlled to a 12 h/12 h light:dark cycle. Two females and one male were placed in a cage together to initiate breeding at D0. At D7, females were weighed to determine if they were pregnant based on weight gain. In addition to vaginal plug detection, a 5.5 g weight gain was correlated with normal pregnancy and embryonic day E11.5 (midgestation), at which point females were housed individually.

Pregnant dams (n = 3–6 per group^40^) from E11.5 through weaning were provided a with water bottles containing either vehicle control (0.5% carboxymethylcellulose; CMC) or CMC plus 31.25 μg/mL DOSS, both prepared in autoclaved 18MOhm water to simulate DOSS use during human pregnancy. Stool softeners (e.g. Docusate, Colace, etc.), which are essentially made of DOSS, are accepted as safe prescription drugs and used by the medical community as the standard of care to treat constipation during pregnancy and breastfeeding^36^. CMC was used to increase palatability of the DOSS solution and has been used as a vehicle control in other obesogen dosing studies^108^. CMC is also commonly found as a component in DOSS-containing stool softeners^109^. The DOSS dosage (31.25 μg/mL) correlates to the dose received by a pregnant woman (88.5 kg average weight, with a daily Docusate sodium stool softener dosage at 5.6 mg/kg = 500 mg DOSS) assuming that an adult mouse weighing 25 g drinks about 4 mL per day. The DOSS dosage given to pregnant dams (31.25 µg/mL) was more than 70-fold less than the no-observed-adverse-effect-level (NOAEL) for maternal DOSS toxicity and teratogenicity set for mice and rats (400 mg/kg) previously^110^. The timing of exposure was based on the incidence of constipation in women becoming elevated from mid-gestation onward^111^. Stock bottles of each solution were prepared as needed (usually weekly). A 1% CMC solution in autoclaved water and a 62.5 μg/mL solution of DOSS in autoclaved water were prepared and then mixed together in a ratio of 1:1 to achieve the final desired concentration for treatment. Autoclaved water was used to dilute the 1% CMC solution 1:1 to achieve the desired concentration of 0.5% CMC for the vehicle control. Only litters with 5–8 pups were used for analysis to control for nutrient availability *in utero* and potential effects of DOSS on litter size. Previous studies indicated that there was no adverse effect on litter size using more than 70-fold higher doses of DOSS^110^. Dosing experiments were repeated three times and control vs. treatment animals from different experiments were grouped for analyses. Investigators were blinded as to the control and treated group samples.

### Body composition assessment

Dual-Energy X-Ray Absorptiometry (DXA; Lunar PIXImus, Madison, WI) was used to assess body composition at 12 weeks of age. Mice were anesthetized using isoflurane, placed into the DXA instrument and scanned individually, ventral down. Data were collected for bone mineral density (BMD; g/cm^2^), bone mineral content (BMC; grams), bone area (cm^2^), lean mass (grams), fat mass (grams) total mass (grams) and percent fat (fat mass/ total mass).

### Animal sacrifice and tissue collection

Animals were humanely sacrificed and exsanguinated at 16 weeks of age. Reproductive fat pads (male epididymal white adipose tissue, EWAT; and female ovarian white adipose tissue, OWAT) and inguinal fat pads (white adipose tissue, IWAT) and livers were dissected, weighed and homogenized (200 mg aliquot in 1 mL Trizol solution) for subsequent nucleic acid isolation. Fat percentage was calculated for each fat pad individually and combined for a representative total fat percentage ((Total WAT mass/16wk body mass) × 100).

### Circulating adipokine and cytokine levels

At 16 weeks, whole blood was collected via cardiac puncture, placed into EDTA coated tubes and incubated at room temperature for at least 15 minutes. All blood collections were performed in the late morning. For plasma collection (500 μL), whole blood was transferred into Eppendorf tubes, centrifuged at 2,000 x g for 15 minutes at 4 °C, aliquoted and stored at −80 °C until analysis.

The following commercially available kits from Meso Scale Discovery (MSD) were used for adiponectin, leptin, insulin, IL-6 and TNF-alpha measurements according to the manufacture’s protocol and using MESO QuickPlex SQ 120 instrumentation: Mouse Adiponectin Kit (K152BXC-1), Mouse Metabolic (leptin and insulin) Kit (K15124C-1) and Mouse Proinflammatory Panel (IL-6 and TNF-alpha) V-PLEX Kit (K15048D). Briefly, each time the assays were run an eight-point standard curve was prepared using calibration standards. Plasma samples were diluted as recommended before measurement. Calibration standards and diluted sample were added to plates in duplicate and processed as recommended. Plates were read on the MSD SECTOR instrument. Discovery Workbench 4.0 Software (Meso Scale Discovery) was used to determine analyte levels based on standard curves.

Plasma C-peptide levels were measured using the Alpco Mouse C-peptide ELISA kit according to the manufacturer’s instructions. Briefly, fasting plasma samples were diluted two fold and glucose stimulated samples were diluted six fold in zero standard solution. Ten microliters of samples, standards and kit controls were added to plated in duplicate and processed as recommended. Stop buffer was added and absorbance at 450 nm was measured using a BioTek plate reader. Standard curves were calculated using a 5 parameter logistic fit. Only concentration values in the range of the standard curve were included in subsequent analyses.

### Gene expression

Homogenized tissue collected in Trizol reagent at the time of sacrifice and stored at −80 °C was used for subsequent nucleic acid isolations. RNA was isolated following the manufacturer’s protocol. For adipose tissue extractions, an additional chloroform wash step was performed to minimalize phenol contamination typically associated with lipid rich tissues. RNA concentration and quality was assessed using Nanodrop ND 1000 (Thermo Fisher Scientific) absorbance ratios 260/280 (2.0–2.2) and 260/230 (>1.5). One μg of RNA was reverse transcribed to cDNA using Superscript III First-Strand Synthesis Super Mix (Invitrogen) following the manufacturer’s instructions. qPCR was performed using the FastStart Essential DNA Green Master Kit (Roche). All primers were designed to be intron spanning to ensure no DNA contamination could be amplified. The same touchdown PCR cycle program was used for all primer pairs: 95 °C 10 sec, 66 °C-56 °C 20 sec dropping a degree each cycle for 10 cycles, then remaining 35 cycles at 56 °C, 72 °C 20 sec for a total of 45 cycles on the LightCycler 96 system (Roche). Results were normalized to the reference gene *Hprt* using the ΔΔCt method as it showed minimal variation between samples across treatment (Ct +/− 0.34 SD). Calibration curves were prepared to ensure equal and high PCR efficiencies (90–110%) between test and reference genes (Supp. Table 1). Data are expressed as a fold change in treated animals compared with control animals.

### Oral glucose tolerance test

At 12 weeks of age, F1 offspring of treated and control dams were assessed for glucose tolerance using an oral glucose tolerance test (oGTT). Before administering the test, animals were fasted for 5–6 hours in cages containing Alpha-Dri bedding with only access to water. At T0, animals were weighed and a baseline blood glucose measurement and plasma sample were taken after making a 0.5–1 mm cut at the tip of the tail with a razor blade. A glucose bolus (2 mg/g body weight) was administered via oral gavage followed with tail tip blood glucose measurements at 15, 30, 60, 90 and 120 minutes. TRUEBalance glucose meter and strips were used for all measurements. If a “HI” error was obtained, the measurement was taken again using a new test strip, and if “HI” was obtained again the value was assigned as 600 (mg/dL), the maximum output of the detector.

### Plasma lipidomic profiling

Lipids were extracted from blood plasma samples using a Bligh-Dyer extraction^112^. Thirty microliters of plasma were spiked with lipid internal standards (36μL), and 4 mL of 1:1 (v:v) chloroform:methanol, followed by the addition of 1.8 mL water. Samples were then incubated on ice for 30 min, vortexed for 20 sec, and centrifuged at 2000 rpm for 10 min to separate organic and aqueous layers. The organic layer was collected and the aqueous layer was re-extracted with 1 mL 1:1 chloroform:methanol. The organic layers were combined, transferred to a new tube, then dried down under nitrogen, and reconstituted in 50 μL of isopropanol.

All lipid extracts were analyzed by ultra-high performance liquid chromatography coupled to high-resolution mass spectrometry (UHPLC-HRMS). Individuals performing extractions and running samples were blinded to sample conditions. Mass spectra were acquired on a Thermo Scientific Orbitrap Fusion Lumos Tribrid mass spectrometer equipped with a heated electrospray ionization (HESI II) probe in positive and negative ion mode. HESI and mass spectrometric parameters for lipid extracts were as follows in positive/negative ion mode, respectively: spray voltage: 3.5/2.5 kV, sheath gas: 40/35 AU; auxiliary nitrogen pressure: 15 AU; sweep gas: 1/0 AU; ion transfer tube and vaporizer temperatures: 325 and 300/275 °C, respectfully; and RF lens level: 30. Full scan, data-dependent MS/MS (top10-ddMS2), and data-independent acquisition mode data were collected at *m/z* 150–2000, corresponding to the mass range of most expected cellular lipids. External calibration was applied before each run to allow for LC-HRMS analysis at 120,000 resolution (at *m/z* 200).

A Thermo Scientific Vanquish UHPLC system (Thermo Scientific, San Jose, CA) was coupled to the Orbitrap Fusion Lumos Tribrid for the chromatographic separation of lipids. The autosampler temperature was maintained at 4 °C for all experiments. Solvent extraction blanks and quality control samples were jointly analyzed over the course of a batch (10–15 samples). A Waters Acquity™ C18 BEH column (2.1 × 100 mm, 1.7 μm particle size, Waters, Milford, MA) maintained at 60 °C was used for all lipidomic studies. The injection volume was 5 μL in positive and negative ion mode with a mobile phase flow rate of 450 μL/min. The gradient program consisted of mobile phase C [60:40 (v:v) acetonitrile/water] and mobile phase D [90:8:2 (v:v:v) isopropanol/ acetonitrile/water], each containing 10 mM ammonium formate and 0.1% formic acid. The gradient included 32% D at 0 min, 40% D at 1 min, a hold at 40% D until 1.5 min, 45% D at 4 min, 50% D at 5 min, 60% D at 8 min, 70% D at 11 min, 80% D at 14 min, 100% D at 16 min, and a hold at 100% D until 17 min. The total run time was 22 min, including a 5 min equilibration.

Full scan raw data files acquired from Xcalibur™ (Thermo Fisher Scientific), centroided and converted to a useable format (mzXML) using MSConvert. Data processing and peak area integration were performed using MZmine, resulting in a feature intensity table. The resulting feature table was filtered using extraction blank samples in FRAMe (Feature Reduction Assistant for Metabolomics v1.0). Only features with a signal/noise ratio greater than 10 times that of the blank were included. LipidSearch™ (Thermo Scientific) and LipidMatch^64^ were used to identify features. Peak areas were normalized to plasma weight for each sample.

### Statistical analysis

We estimated that 12 animals per group would be more than sufficient for this study. This is a conservative number, considering that the Mouse Phenome Database has developed a wealth of phenotypic data, including high-fat diet studies on 43 inbred strains using 7–10 mice per group^113,114^. A sample size of 12 mice per group for comparisons between treatment and control groups provides 80% power to detect a small effect size of 0.44 in obesity outcomes of between treatment groups for a two-sided test at significance level α = 0.004 using a two-sided test and assuming moderate correlation between measures within each mouse (ρ = 0.5). The significance level is Bonferroni adjusted for up to 4 pair-wise comparisons.

For oGTT data, area under the curve (AUC) was calculated for each individual in GraphPad Prism. Means for AUC from treated and control groups were then compared using unpaired student’s *t*-test. Additionally, means for blood glucose were compared at each time point between treated and control groups using two-way ANOVA.

Normality and equal variances were assessed by Shapiro-Wilks test and Brown-Forsythe or F test respectively, before proceeding with mean comparisons using Student’s *t*-test. Data were log transformed if they did not pass the assumptions and to increase normality and Welch’s correction was used to account for differences in variance. Results are discussed in each section and statistical tests are specified in Figure legends. For DNA methylation analysis, average methylation results are determined for each CpG site in an amplicon. Two-way ANOVA was used to assess overall treatment effect and differences observed at specific CpG sites. A p-value of less than 0.05 was considered significant.

## Supplementary information

Supplementary Information

## References

[1] Hales CM, Fryar CD, Carroll MD, Freedman DS, Ogden CL (2018). Trends in Obesity and Severe Obesity Prevalence in US Youth and Adults by Sex and Age, 2007–2008 to 2015-2016. *J*. AMA.

[2] Brown RE (2016). Secular differences in the association between caloric intake, macronutrient intake, and physical activity with obesity. Obes Res Clin Pract.

[3] Speakman JR (2011). Set points, settling points and some alternative models: theoretical options to understand how genes and environments combine to regulate body adiposity. Dis Model Mech.

[4] Menke A, Casagrande S, Geiss L, Cowie CC (2015). Prevalence of and Trends in Diabetes Among Adults in the United States, 1988-2012. JAMA.

[5] Beltran-Sanchez H, Harhay MO, Harhay MM, McElligott S (2013). Prevalence and trends of metabolic syndrome in the adult U.S. population, 1999–2010. J Am Coll Cardiol.

[6] Finkelstein EA (2012). Obesity and severe obesity forecasts through 2030. Am J Prev Med.

[7] Simonson MA, McQueen MB, Keller MC (2014). Whole-genome pathway analysis on 132,497 individuals identifies novel gene-sets associated with body mass index. PLoS One.

[8] Hebebrand J, Volckmar AL, Knoll N, Hinney A (2010). Chipping away the ‘missing heritability’: GIANT steps forward in the molecular elucidation of obesity - but still lots to go. Obes Facts.

[9] Trerotola M, Relli V, Simeone P, Alberti S (2015). Epigenetic inheritance and the missing heritability. Hum Genomics.

[10] Heindel JJ (2017). Metabolism disrupting chemicals and metabolic disorders. Reprod Toxicol.

[11] Maes HH, Neale MC, Eaves LJ (1997). Genetic and environmental factors in relative body weight and human adiposity. Behav Genet.

[12] Waalen J (2014). The genetics of human obesity. Transl Res.

[13] Speliotes EK (2010). Association analyses of 249,796 individuals reveal 18 new loci associated with body mass index. Nat Genet.

[14] Llewellyn CH, Trzaskowski M, Plomin R, Wardle J (2013). Finding the missing heritability in pediatric obesity: the contribution of genome-wide complex trait analysis. Int J Obes (Lond).

[15] Brantley PJ, Myers VH, Roy HJ (2005). Environmental and lifestyle influences on obesity. J La State Med Soc.

[16] Rappaport SM, Smith MT (2010). Epidemiology. Environment and disease risks. Science.

[17] Roseboom TJ (2001). Effects of prenatal exposure to the Dutch famine on adult disease in later life: an overview. Mol Cell Endocrinol.

[18] Stein AD, Lumey LH (2000). The relationship between maternal and offspring birth weights after maternal prenatal famine exposure: the Dutch Famine Birth Cohort Study. Hum Biol.

[19] Baillie-Hamilton PF (2002). Chemical toxins: a hypothesis to explain the global obesity epidemic. J Altern Complement Med.

[20] Grun F, Blumberg B (2006). Environmental obesogens: organotins and endocrine disruption via nuclear receptor signaling. Endocrinology.

[21] Grun F, Blumberg B (2009). Minireview: the case for obesogens. Mol Endocrinol.

[22] Temkin AM (2016). Effects of Crude Oil/Dispersant Mixture and Dispersant Components on PPARgamma Activity *in Vitro* and *in Vivo*: Identification of Dioctyl Sodium Sulfosuccinate (DOSS; CAS #577-11-7) as a Probable Obesogen. Environ Health Perspect.

[23] Iida K, Yonezawa T, Choi SS, Nagai K, Woo JT (2013). Sodium dodecyl sulfate and sodium dodecyl benzenesulfonate are ligands for peroxisome proliferator-activated receptor gamma. J Toxicol Sci.

[24] Kim SH, Park MJ (2014). Phthalate exposure and childhood obesity. Ann Pediatr Endocrinol Metab.

[25] Vafeiadi M (2015). Association of Prenatal Exposure to Persistent Organic Pollutants with Obesity and Cardiometabolic Traits in Early Childhood: The Rhea Mother-Child Cohort (Crete, Greece). Environ Health Perspect.

[26] Mendez MA (2011). Prenatal organochlorine compound exposure, rapid weight gain, and overweight in infancy. Environ Health Perspect.

[27] Burdge GC, Lillycrop KA (2014). Environment-physiology, diet quality and energy balance: the influence of early life nutrition on future energy balance. Physiol Behav.

[28] Hoile SP (2013). Maternal fat intake in rats alters 20:4n-6 and 22:6n-3 status and the epigenetic regulation of Fads2 in offspring liver. J Nutr Biochem.

[29] Lillycrop KA, Burdge GC (2011). The effect of nutrition during early life on the epigenetic regulation of transcription and implications for human diseases. J Nutrigenet Nutrigenomics.

[30] Hines EP (2009). Phenotypic dichotomy following developmental exposure to perfluorooctanoic acid (PFOA) in female CD-1 mice: Low doses induce elevated serum leptin and insulin, and overweight in mid-life. Mol Cell Endocrinol.

[31] EWG. *Environmental Working Group. Skin Deep Cosmetics Database*, http://www.ewg.org/skindeep/ingredient/702082/DIOCTYL_SODIUM_SULFOSUCCINATE/ (2015).

[32] EWG. *Environmental Working Group. Dioctyl Sodium Sulphosuccinate Database Search*, http://www.ewg.org/foodscores/ingredients/9152-DioctylSodiumSulphosuccinate/search (2015).

[33] FDA. *Agency Response Letter GRAS Notice No. GRN 000006*, http://www.fda.gov/Food/IngredientsPackagingLabeling/GRAS/NoticeInventory/ucm154917.htm (1998).

[34] FDA. *Subpart B–Requirements for Specific Standardized Milk and Cream*, http://www.ecfr.gov/cgi-bin/text-idx?SID=27c15e404e137413840ac2dcf2d602af&mc=true&node=pt21.2.131&rgn=div5-se21.2.131_1130 (2015).

[35] Jewell, D. J. & Young, G. Interventions for treating constipation in pregnancy. *The Cochrane database of systematic reviews*, CD001142, 10.1002/14651858.CD001142 (2001). 10.1002/14651858.CD001142 11405974

[36] Mahadevan U, Kane S (2006). American gastroenterological association institute medical position statement on the use of gastrointestinal medications in pregnancy. Gastroenterology.

[37] Trottier M, Erebara A, Bozzo P (2012). Treating constipation during pregnancy. Can Fam Physician.

[38] Hao C, Cheng X, Xia H, Ma X (2012). The endocrine disruptor mono-(2-ethylhexyl) phthalate promotes adipocyte differentiation and induces obesity in mice. Biosci Rep.

[39] Chamorro-Garcia R (2013). Transgenerational inheritance of increased fat depot size, stem cell reprogramming, and hepatic steatosis elicited by prenatal exposure to the obesogen tributyltin in mice. Environ Health Perspect.

[40] Li X, Pham HT, Janesick AS, Blumberg B (2012). Triflumizole is an obesogen in mice that acts through peroxisome proliferator activated receptor gamma (PPARgamma). Environ Health Perspect.

[41] Deng HW, Xu FH, Davies KM, Heaney R, Recker RR (2002). Differences in bone mineral density, bone mineral content, and bone areal size in fracturing and non-fracturing women, and their interrelationships at the spine and hip. J Bone Miner Metab.

[42] Cordey J (1992). Effect of bone size, not density, on the stiffness of the proximal part of normal and osteoporotic human femora. J Bone Miner Res.

[43] Al-Hamodi Z, Al-Habori M, Al-Meeri A, Saif-Ali R (2014). Association of adipokines, leptin/adiponectin ratio and C-reactive protein with obesity and type 2 diabetes mellitus. Diabetol Metab Syndr.

[44] Monteiro, R. & Azevedo, I. Chronic inflammation in obesity and the metabolic syndrome. *Mediators Inflamm* **2010**, 10.1155/2010/289645 (2010). 10.1155/2010/289645 PMC2913796 20706689

[45] Guo J, Jou W, Gavrilova O, Hall KD (2009). Persistent diet-induced obesity in male C57BL/6 mice resulting from temporary obesigenic diets. PLoS One.

[46] Fan Y (2015). Diet-induced obesity in male C57BL/6 mice decreases fertility as a consequence of disrupted blood-testis barrier. PLoS One.

[47] Lihn AS, Pedersen SB, Richelsen B (2005). Adiponectin: action, regulation and association to insulin sensitivity. Obes Rev.

[48] Regnier SM (2015). Dietary exposure to the endocrine disruptor tolylfluanid promotes global metabolic dysfunction in male mice. Endocrinology.

[49] Friedman JM, Halaas JL (1998). Leptin and the regulation of body weight in mammals. Nature.

[50] Maffei M (1995). Leptin levels in human and rodent: measurement of plasma leptin and ob RNA in obese and weight-reduced subjects. Nat Med.

[51] Al Maskari MY, Alnaqdy AA (2006). Correlation between Serum Leptin Levels, Body Mass Index and Obesity in Omanis. Sultan Qaboos Univ Med J.

[52] Surmi BK, Hasty AH (2008). Macrophage infiltration into adipose tissue: initiation, propagation and remodeling. Future Lipidol.

[53] Fried SK, Bunkin DA, Greenberg AS (1998). Omental and subcutaneous adipose tissues of obese subjects release interleukin-6: depot difference and regulation by glucocorticoid. J Clin Endocrinol Metab.

[54] Den Hartigh LJ (2017). Adipocyte-Specific Deficiency of NADPH Oxidase 4 Delays the Onset of Insulin Resistance and Attenuates Adipose Tissue Inflammation in Obesity. Arterioscler Thromb Vasc Biol.

[55] Hsieh PS (2009). COX-2-mediated inflammation in fat is crucial for obesity-linked insulin resistance and fatty liver. Obesity (Silver Spring).

[56] Wong CP, Rinaldi NA, Ho E (2015). Zinc deficiency enhanced inflammatory response by increasing immune cell activation and inducing IL6 promoter demethylation. Mol Nutr Food Res.

[57] Hur K (2003). Aberrant methylation of the specific CpG island portion regulates cyclooxygenase-2 gene expression in human gastric carcinomas. Biochem Biophys Res Commun.

[58] Collins S, Martin TL, Surwit RS, Robidoux J (2004). Genetic vulnerability to diet-induced obesity in the C57BL/6J mouse: physiological and molecular characteristics. Physiol Behav.

[59] Wang CY, Liao JK (2012). A mouse model of diet-induced obesity and insulin resistance. Methods Mol Biol.

[60] Wolfson N, Gavish D, Matas Z, Boaz M, Shargorodsky M (2012). Relation of adiponectin to glucose tolerance status, adiposity, and cardiovascular risk factor load. Exp Diabetes Res.

[61] Aguirre L (2014). Increasing adiposity is associated with higher adipokine levels and lower bone mineral density in obese older adults. J Clin Endocrinol Metab.

[62] Gollisch KS (2009). Effects of exercise training on subcutaneous and visceral adipose tissue in normal- and high-fat diet-fed rats. Am J Physiol Endocrinol Metab.

[63] Markgraf, D. F., Al-Hasani, H. & Lehr, S. Lipidomics-Reshaping the Analysis and Perception of Type 2 Diabetes. *Int J Mol Sci* **17**, 10.3390/ijms17111841 (2016). 10.3390/ijms17111841 PMC5133841 27827927

[64] Koelmel JP (2017). Expanding Lipidome Coverage Using LC-MS/MS Data-Dependent Acquisition with Automated Exclusion List Generation. J Am Soc Mass Spectrom.

[65] Meikle PJ, Christopher MJ (2011). Lipidomics is providing new insight into the metabolic syndrome and its sequelae. Curr Opin Lipidol.

[66] Eisinger K (2014). Lipidomic analysis of serum from high fat diet induced obese mice. Int J Mol Sci.

[67] Barber MN (2012). Plasma lysophosphatidylcholine levels are reduced in obesity and type 2 diabetes. PLoS One.

[68] Hwang LL (2010). Sex differences in high-fat diet-induced obesity, metabolic alterations and learning, and synaptic plasticity deficits in mice. Obesity (Silver Spring).

[69] Pettersson US, Walden TB, Carlsson PO, Jansson L, Phillipson M (2012). Female mice are protected against high-fat diet induced metabolic syndrome and increase the regulatory T cell population in adipose tissue. PLoS One.

[70] Sato H (2014). Sex hormones influence expression and function of peroxisome proliferator-activated receptor gamma in adipocytes: pathophysiological aspects. Horm Mol Biol Clin Investig.

[71] Lecoutre S (2016). Depot- and sex-specific effects of maternal obesity in offspring’s adipose tissue. J Endocrinol.

[72] Rosenfeld CS (2015). Sex-Specific Placental Responses in Fetal Development. Endocrinology.

[73] O’Connell BA, Moritz KM, Walker DW, Dickinson H (2013). Sexually dimorphic placental development throughout gestation in the spiny mouse (Acomys cahirinus). Placenta.

[74] Gabory A (2012). Maternal diets trigger sex-specific divergent trajectories of gene expression and epigenetic systems in mouse placenta. PLoS One.

[75] Gallou-Kabani C (2010). Sex- and diet-specific changes of imprinted gene expression and DNA methylation in mouse placenta under a high-fat diet. PLoS One.

[76] Rabe K, Lehrke M, Parhofer KG, Broedl UC (2008). Adipokines and insulin resistance. Mol Med.

[77] Guo R, Zhang Y, Turdi S, Ren J (2013). Adiponectin knockout accentuates high fat diet-induced obesity and cardiac dysfunction: role of autophagy. Biochim Biophys Acta.

[78] Chand LaS, Serum S (2016). adiponectin level in obese and non obese type 2 dibetes mellitus. International Journal of Clinical and Biomedical Research.

[79] Modan M (1985). Hyperinsulinemia. A link between hypertension obesity and glucose intolerance. J Clin Invest.

[80] Zisman A (2000). Targeted disruption of the glucose transporter 4 selectively in muscle causes insulin resistance and glucose intolerance. Nat Med.

[81] Garvey WT (1998). Evidence for defects in the trafficking and translocation of GLUT4 glucose transporters in skeletal muscle as a cause of human insulin resistance. J Clin Invest.

[82] Kim AY (2015). Obesity-induced DNA hypermethylation of the adiponectin gene mediates insulin resistance. Nat Commun.

[83] Hagman J, Travis A, Grosschedl R (1991). A novel lineage-specific nuclear factor regulates mb-1 gene transcription at the early stages of B cell differentiation. EMBO J.

[84] Konukoglu D, Hatemi H, Bayer H, Bagriacik N (2006). Relationship between serum concentrations of interleukin-6 and tumor necrosis factor alpha in female Turkish subjects with normal and impaired glucose tolerance. Horm Metab Res.

[85] Kirchner H (2014). Altered promoter methylation of PDK4, IL1 B, IL6, and TNF after Roux-en Y gastric bypass. Surg Obes Relat Dis.

[86] Na YK, Hong HS, Lee WK, Kim YH, Kim DS (2015). Increased methylation of interleukin 6 gene is associated with obesity in Korean women. Mol Cells.

[87] Chan PC, Hsiao FC, Chang HM, Wabitsch M, Hsieh PS (2016). Importance of adipocyte cyclooxygenase-2 and prostaglandin E2-prostaglandin E receptor 3 signaling in the development of obesity-induced adipose tissue inflammation and insulin resistance. FASEB J.

[88] Yan Z (2014). Prenatal polycyclic aromatic hydrocarbon, adiposity, peroxisome proliferator-activated receptor (PPAR) gamma methylation in offspring, grand-offspring mice. PLoS One.

[89] Rotter V, Nagaev I, Smith U (2003). Interleukin-6 (IL-6) induces insulin resistance in 3T3-L1 adipocytes and is, like IL-8 and tumor necrosis factor-alpha, overexpressed in human fat cells from insulin-resistant subjects. J Biol Chem.

[90] Simons PJ, van den Pangaart PS, Aerts JM, Boon L (2007). Pro-inflammatory delipidizing cytokines reduce adiponectin secretion from human adipocytes without affecting adiponectin oligomerization. J Endocrinol.

[91] Rai S, Bhatnagar S (2017). Novel Lipidomic Biomarkers in Hyperlipidemia and Cardiovascular Diseases: An Integrative Biology Analysis. OMICS.

[92] van Ginneken, V., Verheij, E., de Vries, E. & van der Greef, J. The Discovery of Two Novel Biomarkers in a High-Fat Diet C56bl6 Obese Mouse Model for Non-Adipose Tissue: A Comprehensive LCMS Study at Hind Limb, Heart, Carcass Muscle, Liver, Brain, Blood Plasma and Food Composition Following a Lipidomics LCMS-Based Approach. *Cellular and Molecular Medicine: Open access* **2** (2016).

[93] Floegel A (2013). Identification of serum metabolites associated with risk of type 2 diabetes using a targeted metabolomic approach. Diabetes.

[94] Kim HJ (2011). Metabolomic analysis of livers and serum from high-fat diet induced obese mice. J Proteome Res.

[95] Nazari M, Kurdi M, Heerklotz H (2012). Classifying surfactants with respect to their effect on lipid membrane order. Biophys J.

[96] Paul HA, Bomhof MR, Vogel HJ, Reimer RA (2016). Diet-induced changes in maternal gut microbiota and metabolomic profiles influence programming of offspring obesity risk in rats. Sci Rep.

[97] Lecomte M (2016). Dietary emulsifiers from milk and soybean differently impact adiposity and inflammation in association with modulation of colonic goblet cells in high-fat fed mice. Mol Nutr Food Res.

[98] Chassaing B (2015). Dietary emulsifiers impact the mouse gut microbiota promoting colitis and metabolic syndrome. Nature.

[99] Akkada AR (1968). Simple method of remove completely ciliate protozoa of adult ruminants. Appl Microbiol.

[100] Yang CM, Varga GA (1993). The effects of continuous ruminal dosing with dioctyl sodium sulphosuccinate on ruminal and metabolic characteristics of lactating Holstein cows. Br J Nutr.

[101] Kim HI (2000). Identification and functional characterization of the peroxisomal proliferator response element in rat GLUT2 promoter. Diabetes.

[102] Lebovitz HE, Banerji MA (2001). Insulin resistance and its treatment by thiazolidinediones. Recent Prog Horm Res.

[103] Mathew J (2012). Dioctyl sulfosuccinate analysis in near-shore Gulf of Mexico water by direct-injection liquid chromatography-tandem mass spectrometry. Journal of chromatography. A.

[104] Flurer, R. A. *et al*. Determination of dioctylsulfosuccinate in select seafoods using a quechers extraction with liquid chromatography- triple quadrupole mass spectrometry. (FDA/ORA/DFS, 2010).

[105] Goodman J, Pang J, Bessman AN (1976). Dioctyl sodium sulfosuccinate- an ineffective prophylactic laxative. J Chronic Dis.

[106] MacMillan TE, Kamali R, Cavalcanti RB (2016). Missed Opportunity to Deprescribe: Docusate for Constipation in Medical Inpatients. Am J Med.

[107] Fraulob JC, Ogg-Diamantino R, Fernandes-Santos C, Aguila MB, Mandarim-de-Lacerda CA (2010). A Mouse Model of Metabolic Syndrome: Insulin Resistance, Fatty Liver and Non-Alcoholic Fatty Pancreas Disease (NAFPD) in C57BL/6 Mice Fed a High Fat Diet. J Clin Biochem Nutr.

[108] Kirchner S, Kieu T, Chow C, Casey S, Blumberg B (2010). Prenatal exposure to the environmental obesogen tributyltin predisposes multipotent stem cells to become adipocytes. Mol Endocrinol.

[109] Dujovne CA, Shoeman DW (1972). Toxicity of a hepatotoxic laxative preparation in tissue culture and excretion in bile in man. Clin Pharmacol Ther.

[110] Fiume, M. M. *et al*. In *Cosmetic Ingredient Review Expert Panel Meeting Cosmetic Ingredient Review Panel* 29 (Cosmetic Ingredient Review 1101 17th Street, NW, Suite 412, Washington, DC 20036-4702; ph 202.331.0651; fax 202.331.0088; cirinfo@cir-safety.org, 2013).

[111] Johnson P, Mount K, Graziano S (2014). Functional bowel disorders in pregnancy: effect on quality of life, evaluation and management. Acta Obstet Gynecol Scand.

[112] Bligh EG, Dyer WJ (1959). A rapid method of total lipid extraction and purification. Can J Biochem Physiol.

[113] Svenson KL (2007). Multiple trait measurements in 43 inbred mouse strains capture the phenotypic diversity characteristic of human populations. J Appl Physiol (1985).

[114] Shockley KR, Witmer D, Burgess-Herbert SL, Paigen B, Churchill GA (2009). Effects of atherogenic diet on hepatic gene expression across mouse strains. Physiol Genomics.

