# Supplementary material for: Increased adiposity, inflammation, metabolic disruption and dyslipidemia in adult male offspring of DOSS treated C57BL/6 dams
Source: Sci Rep. 2019 Feb 6;9:1530. doi: 10.1038/s41598-018-38383-9 (PMC6365642; doi:10.1038/s41598-018-38383-9)
Supplement: Supplementary file 1 — Supplementary Information [file 41598_2018_38383_MOESM1_ESM.pdf]

Increased adiposity, inflammation, metabolic disruption and dyslipidemia in adult male offspring of DOSS treated C57BL/6 dams

Alexis M. Temkin[1], Robert R. Bowers[2], Candice Z. Ulmer[3], Kayla Penta[4], John A. Bowden[3], Jennifer Nyland[5], John E. Baatz[6], and *Demetri D. Spyropoulos[1,2]

[1]Molecular and Cellular Biology and Pathobiology Program, Medical University of South Carolina, Charleston, SC; [2]Department of Pathology and Laboratory Medicine, Medical University of South Carolina, Charleston, SC; [3]National Institute of Standards and Technology, Chemical Sciences Division, Hollings Marine Laboratory, Charleston, SC; [4]Department of Biological Sciences, University of South Carolina, Columbia, SC; [5]Department of Biological Sciences, Salisbury University, Salisbury, MD; [6]Department of Pediatrics and Neonatology, Medical University of South Carolina, Charleston, SC

*Address correspondence to D.D. Spyropoulos, Pathology and Laboratory Medicine, MUSC, Darby Children's Research Institute, CRI 208, 173 Ashley Ave., Charleston, SC 29425 USA. Telephone: (843) 792-1625. E-mail: spyrodd@musc.edu

Supplemental Figures:

Legends:

Supp Figure 1. Body composition at 12 weeks of age in F1 male and female mice treated with DOSS and vehicle control.

DXA scans were used to assess body composition in male and female mice at 12 weeks of age (males, n = 12/group; females, n = 16/group). Measurements were obtained for A) bone mineral density and B) bone mineral content. Graph bars represent means and standard deviations. (Male and female mice were analyzed separately using unpaired *t*-test *$p<0.05$).

Supp Figure 2. Developmental DOSS treatment promotes a proinflammatory state in adult F1 male mice.

Plasma and adipose tissue were collected at time of sacrifice (16 weeks; n = 12/group). Gene expression was determined via RNA isolation, cDNA conversion and qPCR using the delta delta Ct method with *Hprt* as the housekeeping gene. Results are shown for A) plasma IL-6 correlated with fat mass, B) IL-6 gene expression in IWAT tissue (*$p<0.05$ unpaired *t*-test). Graph bars represent means and standard deviations.

Supp Figure 3. DOSS promotes changes in DNA methylation in promoter regions of inflammatory genes.

Targeted bisulfite sequencing was used to assess promoter methylation in IWAT tissue for genes associated with increased gene expression or circulating protein levels upon DOSS treatment. Results were obtained for: A) IL-6 promoter methylation, B) IL-6

CpG2 percent methylation correlated with circulating IL-6 levels, C) Cox2 promoter methylation and D) Cox2 CpG1 percent methylation (n = 12/group). Two-Way Anova was used to assess significant changes in DNA methylation based on treatment across the whole promoter region with Sidak's post-hoc test for individual CpG sites differences (*p<0.05 treated vs. control, Two-Way Anova). Graph bars represent means and standard deviations. Individual sites were then also compared using Sidak's post-hoc test (#p<0.05). Linear regression was used to determine correlations between CpG2 percent methylation and circulating IL-6 levels.

Supp Figure 4. Effects of DOSS treatment on promoter methylation and relationships to gene expression.

Targeted bisulfite sequencing was used to assess promoter methylation in IWAT tissue for genes associated with increased gene expression or circulating protein levels upon DOSS treatment (n = 12/group). Results were obtained for: A) IL-6 CpG2 percent methylation correlation with *IL-6* IWAT gene expression, B) Cox2 CpG1 percent methylation correlated with *Cox2* IWAT expression, C) adiponectin region 1 promoter methylation, D) adiponectin region 2 promoter methylation, and E) adiponectin region 2 CpG3 percent methylation correlated with *AdipoQ* IWAT gene expression. Two-Way Anova was used to assess significant changes in DNA methylation based on treatment across the whole promoter region with Sidak's post-hoc test for individual CpG sites differences (*p<0.05 DOSS treated vs. control, Two-Way Anova). Graph bars represent means and standard deviations. Individual sites were then also compared using Sidak's post-hoc test (#p<0.05). Linear regression was used to determine correlations between percent methylation and gene expression.

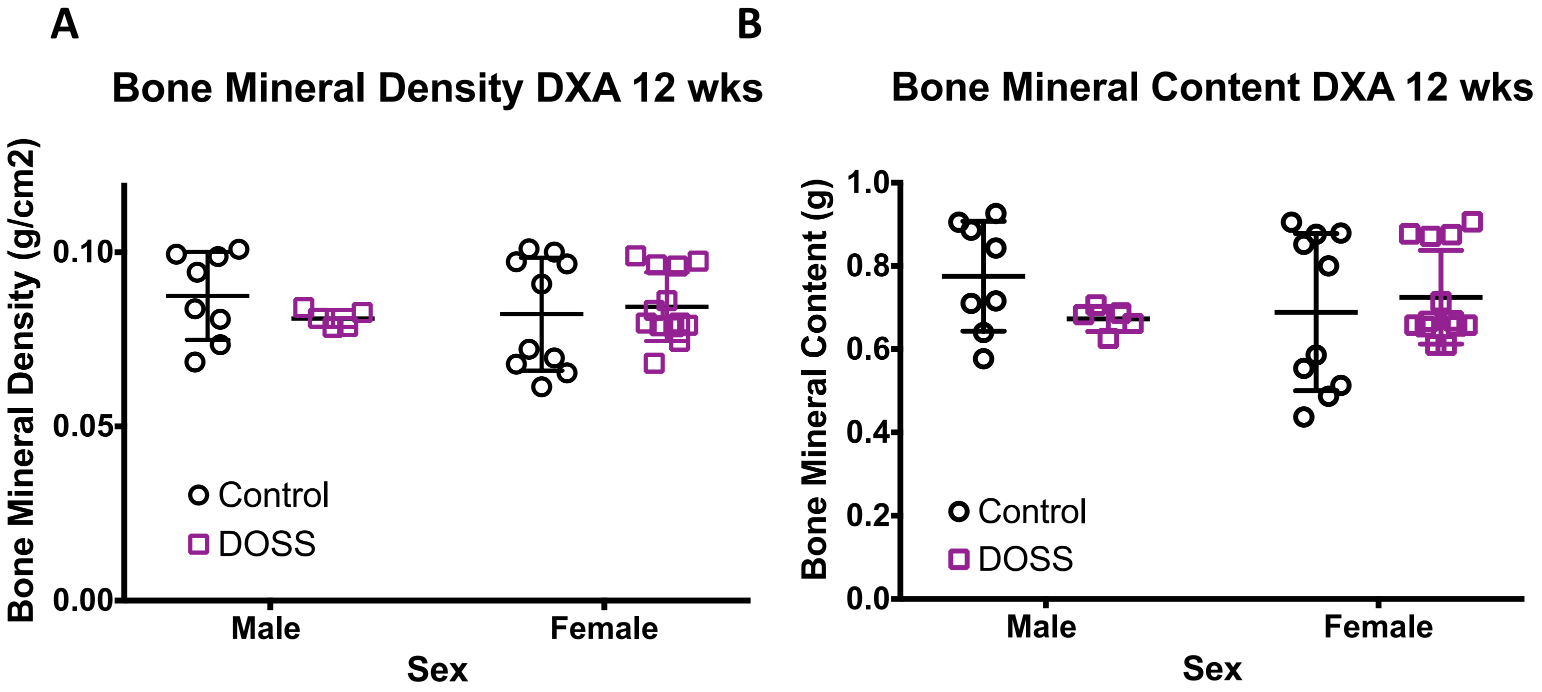

Supp. Figure 1.

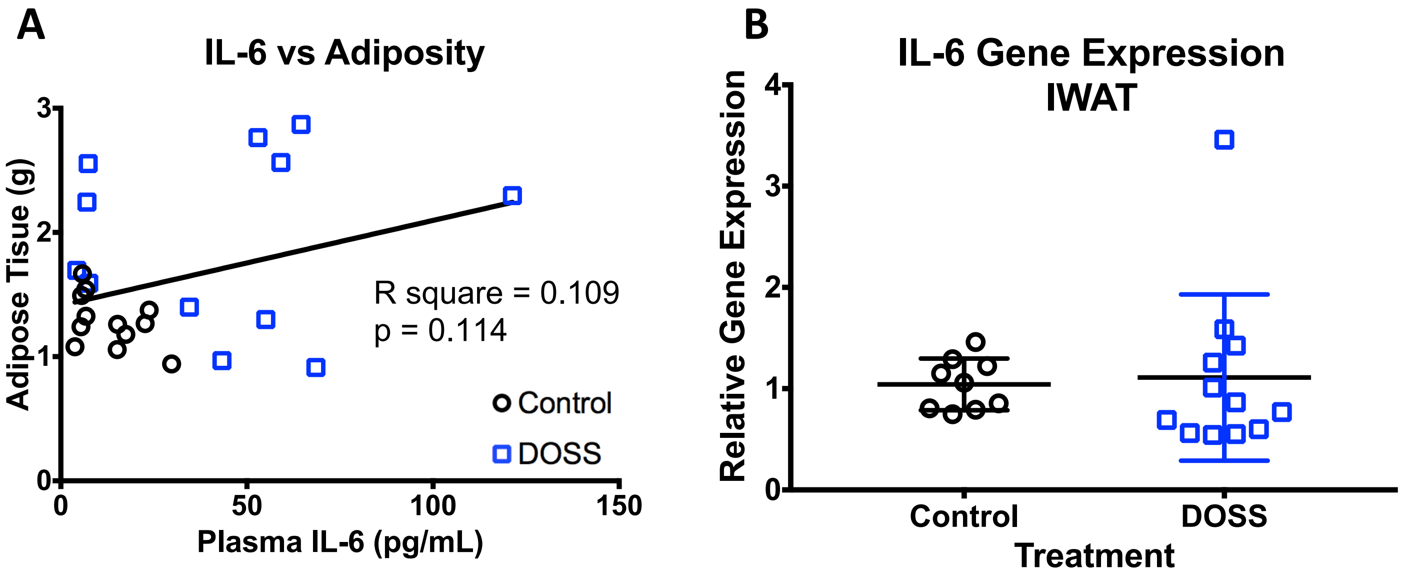

Supp. Figure 2.

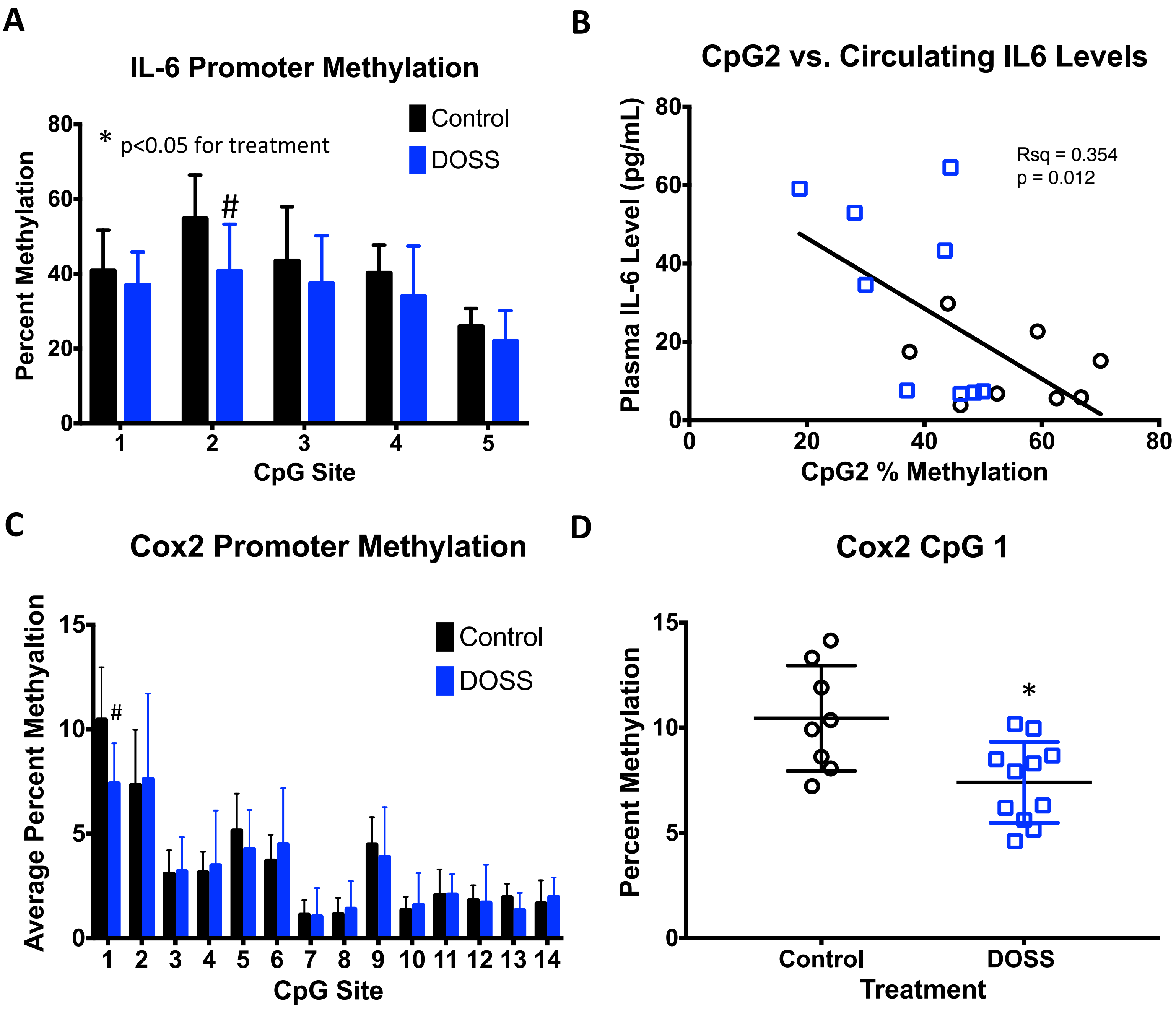

Supp. Figure 3.

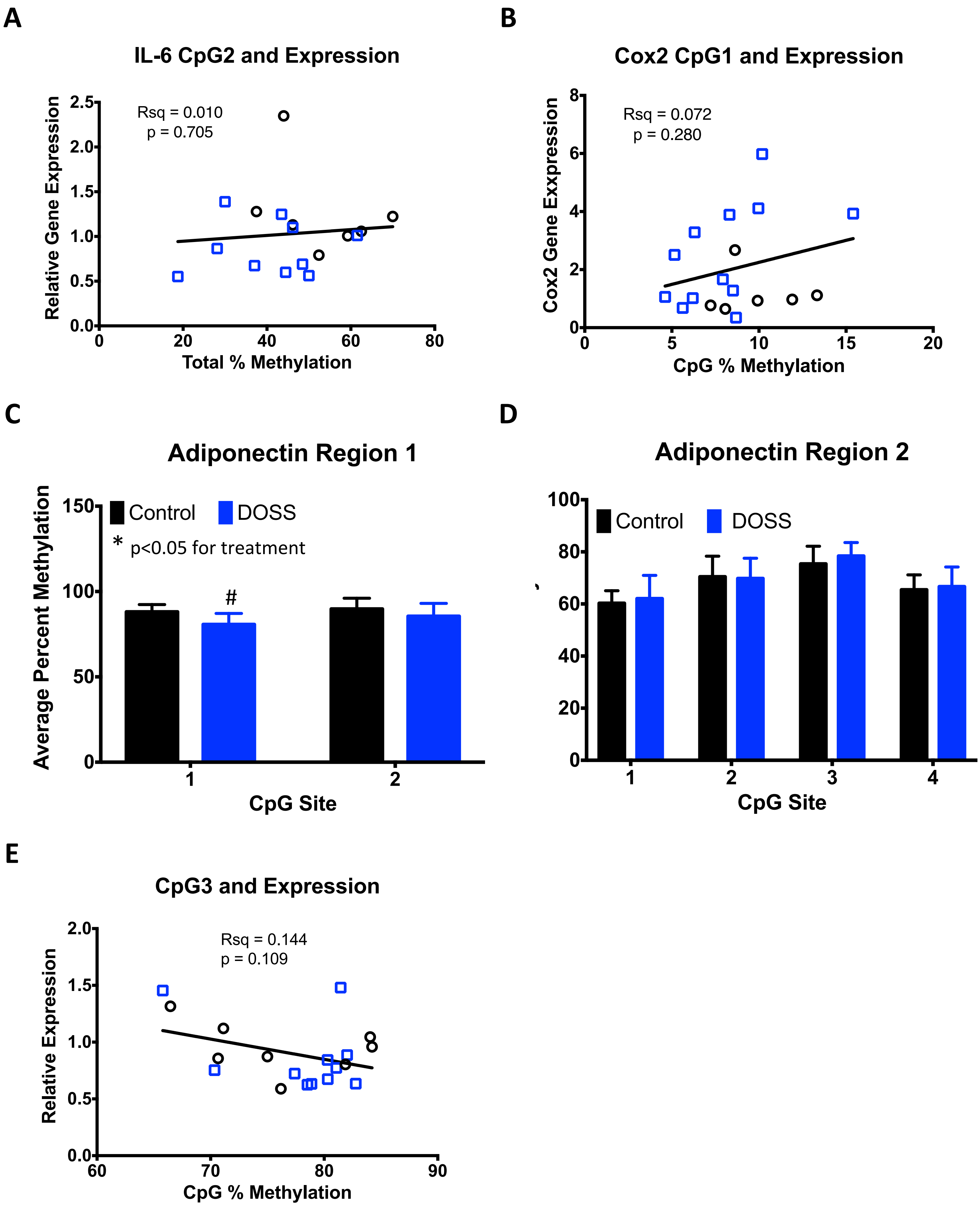

Supp. Figure 4.

Supplemental Methods:

DNA Methylation analysis via targeted bisulfite sequencing

DNA Isolation

After RNA was isolated from Trizol reagent samples, residual interphase and organic material was stored at 4°C until DNA isolation was performed. Samples were centrifuged at 15,000 x g at 4°C for 15 minutes. Any remaining aqueous phase containing RNA was removed. An equal volume (about 600μL) of Back Extraction Buffer (4 M guanidine thiocyanate, 50 mM sodium citrate and 1 M Tris) was added to interphase-organic phase mixture. The samples were vigorously mixed by inversion an incubated at room temperature for 10 minutes. Samples were centrifuged at 15,000 x g at 4°C for 15 minutes and 420uL of the aqueous phase was transferred to a separate tube. At this point samples were processed following a modified protocol for Qiagen Blood and Tissue DNeasy Kit. Briefly, 200uL of ethanol was added to each sample, mixed well and pipetted onto a provided spin column, centrifuged and flow through discarded. The column was washed 1x with 500uL Buffer AW1, then 1X with 500uL Buffer AW2. Care was taken to ensure the column was completely free of ethanol before eluting DNA with 75uL Buffer AE. DNA concentrations were quantified using Nanodrop ND 1000 (ThermoFisher Scientific).

Bisulfite Conversion and PCR Amplification

Genomic DNA (500ng) from IWAT of 16 week-old mice was bisulfite converted for methylation analysis using the Methylamp DNA Modification Kit (Epigentek) following the manufacturer's protocol. Eight control samples and 12 treated samples were used and selected based on their DNA quality. Target genes for PCR were selected based on previous studies that identified methylation or expression changes in these genes as a result of obesity, glucose

intolerance and diabetes, or obesogen exposure (Supp Table 2). Studies from both human and mice were utilized. For targets where primers were listed in publications, these primers were used with conditions optimized in-house. For new targets, primers were designed using MethPrimer in promoter regions of genes flanking CpG islands. For PCR amplification, 12.5 ng of converted DNA was used with EpiMark HotStart Taq DNA polymerase following the manufacturer's recommendations (Supp Table 2). Amplification of single products was validated using gel electrophoresis. PCR products were purified using GenCatch PCR Clean Up Kit (Epoch) and quantified using Nanodrop equipment (Thermo Fisher Scientific). All products from a single individual were then pooled using equal nanomoles with volumes adjusted based on nM concentration (based on lowest concentration) for sequencing.

Illumina Library Prep, Next Generation Sequencing and Analysis

The MUSC Cancer Genomics core was used for next generation sequencing of products. The sequencing library was prepared using the TruSeq Kit, following the manufacturer's protocol beginning at "End Repair." Adapters were ligated so each individual was barcoded. Samples were then paired end sequenced on an Illumina MiSeq. All data analysis was performed in BaseSpace. Raw sequences were trimmed using Trimmomatic [1]. Methylation analysis was performed in Methyl Seq v1.0 (Illumina, Inc.). Methyl Seq aligns generated sequences to a bisulfite converted genome using Bowtie 2 [2]. Sequences were aligned to Mouse mm9 using a targeted manifest specifically for targeted amplicons based on chromosomal locations for start and end sites. Since this was a targeted analysis, the differences between mm9 and mm10 likely would not impact differences in sequence alignment. CpG methylation status was obtained for each CpG in an amplicon using BisMark [3]. If a sample did not have at least 12 reads per CpG

site it was dropped from analysis. No correlation was observed between read count and methylation status (data not shown).

Supplemental Tables:

Table 1. Genes and corresponding primers used for qRT - PCR analysis

Gene	Forward Primer	Reverse Primer
Hprt	AGGCCAGACTTTGTTGGATTTG	TTCAACTTGCGCTCATCTTAGG
AdipoQ	GTTCCTCTTAATCCTGCCCA	CTCCTGTCATTCCAACATCTC
Leptin	CCTGTGTCGGTTCCTGTG	CCTGTTGATAGACTGCCAGAG
IL-6	AGCCAGAGTCCTTCAGAGAGAT	GAGAGCATTGGAAATTGGGGT
Cox2	TTCAACACACTCTATCACTGGC	AGAAGCGTTTGCGGTACTCAT
Nox4	CCTTTTACCTATGTGCCGGAC	CATGTGATGTGTAGAGTCTTGCT

Table 2. Bisfulfite sequencing primer sequences, chromosomal location, product size, CpG number and corresponding references.

Gene	Forward	Reverse	Chromosomal location (mm9)	Size (bp)	CpG #	Reference	Promoter methylation status associated with gene expression (Y/N)
**AdipoQ R1**	AGGTAAGTGTTTTGTGATATTGGGT	ACACCCACAATAATTCCATAAAATC	chr16:23145842+23146117	276	2	[4]	No
**AdipoQ R2**	TGGAGGAAGTAGATGTTTGGTTAGT	CAAAACAATACCTTAAAAACCTCTC	chr16:23145441+23145636	196	4	[4]	Yes
**IL-6**	TGTTTAGGTTGGGTGTTG	ACCCTAAAAAACATAAACACTCTTC	chr5:30339113+30339453	341	5	[5]	Yes
**Cox-2**	AGATGTGGATTTTGATAGAGGATATT	CTACCCTTAACTACCCCAAATAATAC	chr1:151946703+151947035	333	14	[6]	Yes
**Leptin**	GAGTAGTTAGGTTAGGTATGTAAAGAG	TAATAACTACCCCAATACCACTTAC	chr6:29009816+29010194	379	19	[7]	Yes, but inconsistent results
**Fabp4**	AGGAATTGTTTTTTTTGAAAAGTAG	AATAAAACACCTCCAAACACTATACC	chr3:10202723+10202992	270	5	[8]	Yes
**Glut4**	TGGGTTATATGTATTTGTTAGGGTA	TATTAATCCCTTAAATCATCTCCTC	chr11:69761972+69762232	261	13	[9]	Unknown
**Fasn**	TAGTAGGTAGGATAGGGAATATTGA	CAACCTCTCTAAACACTCAAAAAAC	chr11:120686159+120686445	286	17	[10]	Evidence in rats
**Irs-1**	GTAGTGGGTTTAGGGTGAGTGTAGT	CCCCTACCCAAAAATATTTAATTTAC	chr1:82287290+82287526	237	15	[11,12]	Yes for a similar region in humans
**Hmox1**	GGGTTGGATGTTGTAATAGTAG	CATTCCCAAACAAAATAAAAAACAC	chr8:77617422+77617730	308	24	[13]	Unknown
**Pparg2**	GATGTGTGATTAGGAGTTTTAATTAAAG	CAAACCTAAATTAACTAACACTATCCTAAC	chr6:115371595+115371953	259	4	[14]	Yes

Table 3. Average percent methylation of interrogated genes in IWAT tissue of Control and DOSS males.

**Gene Loci**	**Percent Methylation** Control (Mean +/- SD)	**Percent Methylation** DOSS (Mean +/- SD)	**P value: 2-Way Anova Treatment/Post-Hoc CpG**	**Coverage** (Mean +/- SE)
**AdipoQ R1**	88.93 +/- 4.8	83.13 +/- 6.6	**0.008/0.032**	121.5 +/- 45.3
**AdipoQ R2**	67.88 +/- 5.7	69.21 +/- 6.2	0.431/NA	155.6 +/- 44.3
**IL-6**	41.10 +/- 7.4	34.29+/-9.3	**0.004**/**0.041**	41.5 +/- 15.5
**Cox-2**	3.46 +/- 0.5	3.30 +/- 0.3	0.349/**0.005**	280.6 +/- 69.7
**Leptin**	64.08 +/- 4.8	65.06 +/- 6.4	0.240/NA	141.4 +/- 56.3
**Fabp4**	78.01 +/- 5.8	77.46 +/- 11.9	0.789/NA	128.6 +/-32.3
**Glut4**	3.01 +/- 0.5	3.02+/- 0.9	0.996/NA	136.0 +/- 41.5
**Fasn**	2.66 +/- 0.3	2.73 +/- 0.7	0.752/NA	113.2 +/-41.6
**Irs-1**	3.37 +/- 0.5	2.28 +/- 0.5	0.631/NA	237.9 +/-41.6
**Hmox1**	1.66 +/- 0.2	1.59 +/- 0.4	0.535/NA	211.4 +/- 38.7
**Pparg2**	63.83 +/- 6.7	61.69 +/- 12.4	0.412/NA	127.5 +/- 36.9

References Cited:

1 Bolger, A. M., Lohse, M. & Usadel, B. Trimmomatic: a flexible trimmer for Illumina sequence data. *Bioinformatics* **30**, 2114-2120, doi:10.1093/bioinformatics/btu170 (2014).

2 Langmead, B. & Salzberg, S. L. Fast gapped-read alignment with Bowtie 2. *Nat Methods* **9**, 357-359, doi:10.1038/nmeth.1923 (2012).

3 Krueger, F. & Andrews, S. R. Bismark: a flexible aligner and methylation caller for Bisulfite-Seq applications. *Bioinformatics* **27**, 1571-1572, doi:10.1093/bioinformatics/btr167 (2011).

4 Kim, A. Y. *et al.* Obesity-induced DNA hypermethylation of the adiponectin gene mediates insulin resistance. *Nat Commun* **6**, 7585, doi:10.1038/ncomms8585 (2015).

5 Wong, C. P., Rinaldi, N. A. & Ho, E. Zinc deficiency enhanced inflammatory response by increasing immune cell activation and inducing IL6 promoter demethylation. *Mol Nutr Food Res* **59**, 991-999, doi:10.1002/mnfr.201400761 (2015).

6 Domingo-Gonzalez, R., Huang, S. K., Laouar, Y., Wilke, C. A. & Moore, B. B. COX-2 expression is upregulated by DNA hypomethylation after hematopoietic stem cell transplantation. *J Immunol* **189**, 4528-4536, doi:10.4049/jimmunol.1201116 (2012).

7 Shen, W. *et al.* Epigenetic modification of the leptin promoter in diet-induced obese mice and the effects of N-3 polyunsaturated fatty acids. *Sci Rep* **4**, 5282, doi:10.1038/srep05282 (2014).

8 Kirchner, S., Kieu, T., Chow, C., Casey, S. & Blumberg, B. Prenatal exposure to the environmental obesogen tributyltin predisposes multipotent stem cells to become adipocytes. *Mol Endocrinol* **24**, 526-539, doi:10.1210/me.2009-0261 (2010).

9 Giacchetti, G. *et al.* Decreased expression of insulin-sensitive glucose transporter mRNA (GLUT-4) in adipose tissue of non-insulin-dependent diabetic and obese patients: evaluation by a simplified quantitative PCR assay. *J Endocrinol Invest* **17**, 709-715, doi:10.1007/BF03347765 (1994).

10 Gracia, A. *et al.* Fatty acid synthase methylation levels in adipose tissue: effects of an obesogenic diet and phenol compounds. *Genes & nutrition* **9**, 411, doi:10.1007/s12263-014-0411-9 (2014).

11 Cortese, R., Khalyfa, A., Bao, R., Andrade, J. & Gozal, D. Epigenomic profiling in visceral white adipose tissue of offspring of mice exposed to late gestational sleep fragmentation. *International journal of obesity* **39**, 1135-1142, doi:10.1038/ijo.2015.38 (2015).

12 Rohde, K. *et al.* IRS1 DNA promoter methylation and expression in human adipose tissue are related to fat distribution and metabolic traits. *Sci Rep* **7**, 12369, doi:10.1038/s41598-017-12393-5 (2017).

13 Jais, A. *et al.* Heme oxygenase-1 drives metaflammation and insulin resistance in mouse and man. *Cell* **158**, 25-40, doi:10.1016/j.cell.2014.04.043 (2014).

14 Fujiki, K., Kano, F., Shiota, K. & Murata, M. Expression of the peroxisome proliferator activated receptor gamma gene is repressed by DNA methylation in visceral adipose tissue of mouse models of diabetes. *BMC Biol* **7**, 38, doi:10.1186/1741-7007-7-38 (2009).
